# Roux-En-Y Gastric Bypass (RYGB) Surgery during High Liquid Sucrose Diet Leads to Gut Microbiota-Related Systematic Alterations

**DOI:** 10.3390/ijms23031126

**Published:** 2022-01-20

**Authors:** Laimdota Zizmare, Christina N. Boyle, Sabrina Buss, Sandrine Louis, Laura Kuebler, Ketki Mulay, Ralf Krüger, Lara Steinhauer, Isabelle Mack, Manuel Rodriguez Gomez, Kristina Herfert, Yvonne Ritze, Christoph Trautwein

**Affiliations:** 1 Werner Siemens Imaging Center, Department of Preclinical Imaging and Radiopharmacy, University Hospital Tübingen, Eberhard Karls University of Tübingen, Röntgenweg 13, 72076 Tübingen, Germany; Laimdota.Zizmare@med.uni-tuebingen.de (L.Z.); Sabrina.Buss@med.uni-tuebingen.de (S.B.); Laura.Kuebler@med.uni-tuebingen.de (L.K.); Kristina.Herfert@med.uni-tuebingen.de (K.H.); Christoph.Trautwein@med.uni-tuebingen.de (C.T.); 2 Institute of Veterinary Physiology, University of Zurich, Winterthurerstraße 260, CH-8057 Zurich, Switzerland; boyle@vetphys.uzh.ch; 3 Department of Physiology and Biochemistry of Nutrition, Max Rubner-Institut, Haid-und-Neu-Straße 9, 76131 Karlsruhe, Germany; Sandrine.Louis@mri.bund.de (S.L.); Ralf.Krueger@mri.bund.de (R.K.); Lara.Steinhauer@mri.bund.de (L.S.); Manuel.RodriguezGomez@mri.bund.de (M.R.G.); 4 Department of Medical Psychology and Behavioral Neurobiology, Eberhard Karls University of Tübingen, Silcherstraße 5, 72076 Tübingen, Germany; mulay.ketki@yahoo.co.in; 5 Department of Psychosomatic Medicine and Psychotherapy, University Hospital Tübingen, Osianderstraße 5, 72076 Tübingen, Germany; Isabelle.Mack@uni-tuebingen.de

**Keywords:** RYGB, metabolomics, microbiome, inflammation, gut-brain axis, GABA, 3-hydroxybutyrate

## Abstract

Roux-en-Y gastric bypass (RYGB) surgery has been proven successful in weight loss and improvement of co-morbidities associated with obesity. Chronic complications such as malabsorption of micronutrients in up to 50% of patients underline the need for additional therapeutic approaches. We investigated systemic RYGB surgery effects in a liquid sucrose diet-induced rat obesity model. After consuming a diet supplemented with high liquid sucrose for eight weeks, rats underwent RYGB or control sham surgery. RYGB, sham pair-fed, and sham ad libitum-fed groups further continued on the diet after recovery. Notable alterations were revealed in microbiota composition, inflammatory markers, feces, liver, and plasma metabolites, as well as in brain neuronal activity post-surgery. Higher fecal 4-aminobutyrate (GABA) correlated with higher Bacteroidota and *Enterococcus* abundances in RYGB animals, pointing towards the altered enteric nervous system (ENS) and gut signaling. Favorable C-reactive protein (CRP), serine, glycine, and 3-hydroxybutyrate plasma profiles in RYGB rats were suggestive of reverted obesity risk. The impact of liquid sucrose diet and caloric restriction mainly manifested in fatty acid changes in the liver. Our multi-modal approach reveals complex systemic changes after RYGB surgery and points towards potential therapeutic targets in the gut-brain system to mimic the surgery mode of action.

## 1. Introduction

Obesity is a metabolic and inflammatory health condition characterized by excess accumulation of body fat, which increases the risk of developing an unbalanced, unresolved metabolic inflammation within adipose tissue and in metabolic organs such as the liver, pancreas and, brain [1,2]. A chronic, whole-body meta-inflammatory state during obesity can further lead to cardiovascular disease, type 2 diabetes, dysregulation of the immune system, and impaired cognitive and mental capabilities [3,4,5,6,7,8]. Prolonged intake of high sugar foods, a principal component of the Western diet, promoted excess energy intake and gain of body weight [9]. Moreover, increased sugar intake leads to gut microbiota adaptations and dysbiosis [10]. Alongside cardiovascular complications, obesity and type 2 diabetes are important risk factors in the pathogenesis of cognitive dysfunction [11,12,13]. On the cellular level, a Western diet is associated with reduced expression of brain-derived neurotrophic factors, elevated levels of oxidative stress, and pro-inflammatory processes in neurons, and affects the whole-body immune response [14].

Considering that obesity has become a global pandemic [15], effective prevention and treatment strategies are needed. This is especially urgent for the 39 million children under the age of five diagnosed as overweight or obese in 2020 [16]. Beyond classical dietary and drug treatment [17,18], currently available approaches to treat morbid obesity and type 2 diabetes include bariatric surgery [19].

Roux-en-Y gastric bypass (RYGB) surgery is the most frequently employed bariatric technique in Western countries [20]. The surgical intervention consists of transection of the stomach, leaving a small gastric pouch, which is anastomosed to a distal part of the small intestine, creating a Roux (dietary) limb [21]. The rearrangement of the gut allows ingested food direct access to the small intestinal lumen, where it is eventually joined with the biliopancreatic limb, from which point the common channel is formed. After RYGB surgery, patient preferences for high-carbohydrate and high-fat foods decreased [22,23], and patients reportedly lost the motivation to eat [22]. Similarly, the preference for a high-fat diet steadily decreased, and the preference for a standard low-fat chow increased over a five-month post-surgical period in a rat model of RYGB [24].

RYGB surgery improves metabolic health and diabetes remission more effectively than other treatment strategies, including pharmacotherapy and lifestyle interventions [25,26]. Several murine RYGB studies show a positive impact of the surgery on various metabolic parameters reducing the risk of type 2 diabetes mellitus [27,28,29] and hyperlipidemia [30]. RYGB surgery leads to reduced glycemia, as shown by functional studies of the liver and brain. Surgery led to an improvement in liver health and glycemic control, further reducing the risk of hepatic steatosis [31]. RYGB surgery was also reported to lower lipogenesis and increase fatty acid beta-oxidation in the type 2 diabetes rat model [31]. Moreover, indications of lowered glycemia were also reported by a positron emission tomography (PET) imaging study where increased neuronal activity was shown in the hypothalamic and thalamic brain regions [13].

Fundamental changes in the gut microbiota and related metabolites are another common RYGB surgery downstream effect. *Escherichia coli*, *Klebsiella pneumonia*, *Streptococcus*, *Bifidobacterium dentium,* and *Akkermansia muciniphila* have been reported in an increased abundance in patient feces after RYGB surgery [32]. Murine model studies have revealed a highly affected microbiome by RYGB surgery and have further shown a direct microbiota impact on weight and fat reduction after feces transplantation due to maintained increase of *Escherichia* and *Akkermansia* species abundance [33]. Gut microbiota dysbiosis has also been previously linked to obesity and food addiction due to interactions along the gut-brain axis via altered inflammatory signals such as tumor necrosis factor (TNF), interleukin (IL)-6, IL-1 beta, plasma lipopolysaccharide (LPS) [34], and neuroactive metabolites [35].

Metabolic profiling of RYGB surgery patients is a prospectively valuable tool for identifying subjects with type 2 diabetes that would have the most beneficial surgery outcome [36]. Murine metabolomics studies in RYGB models and patients have reported common altered fecal metabolite patterns such as upregulated trimethylamine (TMA), trimethylamine N-oxide (TMAO), glycine, 4-aminobutyrate (GABA), and downregulated plasma branched-chain amino acids (BCAA), arginine, and the tryptophan-kynurenine pathway [37,38]. However, further studies in animal models are crucial for appropriate biomarker discovery and their translation to clinically relevant sampling such as plasma or serum, feces, and urine [39].

A significant portion of currently available publications has investigated the impact of RYGB in genetic models of obesity or diet-induced obesity models resulting from high-fat solid diets. Excessive caloric intake, often in a form of artificial sweetened soft drinks and juices, is one of the causes leading to obesity among children and adolescents [40]. Similarly, access to a liquid high sucrose diet increases caloric intake in mice [41] and, therefore, enhances obesity progression and alters the body’s metabolism. It has also been shown that a high solid sucrose diet, similarly to a high-fat diet, promoted liver damage and affected insulin signaling even when the serum insulin levels were not changed [31]. However, the metabolic impact of RYGB surgery in an obesity model resulting from liquid high sucrose intake has not yet been described.

In this exploratory study, we examined the effects of surgery, caloric restriction, and liquid sucrose diet in RYGB-operated (RYGB group) versus sham-operated pair-fed (PF) and *ad libitum*-fed (AdLib) rats. We investigated gut microbiota dysbiosis, fecal and plasma metabolites, liver fatty acid composition, inflammatory immune marker correlations, and brain glucose metabolism for a comprehensive, multi-modal characterization of liquid sucrose-induced obesity. This systemic multi-modal characterization of the detailed molecular effects following RYGB surgery will help develop novel potential therapeutics that mimic the invasive surgical approach.

## 2. Results

### 2.1. RYGB Surgery Leads to Reduced Body Weight, Food, and Liquid Sucrose Intake

**Figure 1:**
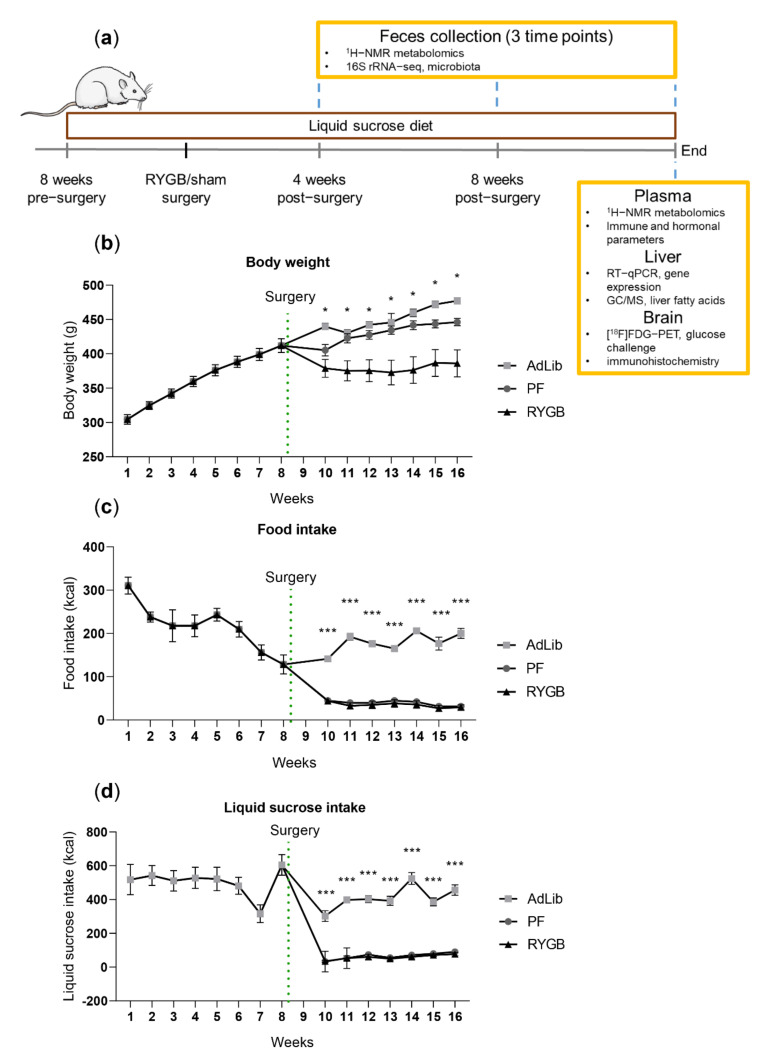
Overview of the experimental setup before and after the Roux-en-Y gastric bypass (RYGB) surgery. The timeline of the experiments, including sample collection points and analytical techniques (**a**). Average body weight (**b**), food intake (kcal) (**c**), and liquid sucrose intake (kcal) (**d**) are shown for *ad libitum* (AdLib), pair-fed (PF), and Roux-en-Y gastric bypass-operated (RYGB) rats. Data shown as mean ± SEM, *p*-values shown for RYGB vs. AdLib comparison. *** < 0.001, * < 0.05, two-way ANOVA, Tukey’s multiple comparisons test. RYGB (*n* = 5) black triangles, PF (*n* = 8) dark grey dots, AdLib (*n* = 7) light grey squares.

After an initial eight-week period of liquid sucrose diet and weight gain, rats underwent RYGB or sham surgery, followed by a second eight-week phase of liquid sucrose feeding after recovery (Figure 1a). We monitored the development of body weight, food, and liquid sucrose intake (Figure 1b–d). RYGB group sustained a significant weight loss compared to AdLib animals (*p* < 0.05) (Figure 1b). PF and RYGB animals showed similar weight loss at week 10 of the experiment (week 2 post-surgery). By week 11, PF animals had gained weight similar to the AdLib rats, while RYGB rats remained lean. RYGB animals had heterogeneous eating habits consuming between 84–105 kcal/day for the final six weeks before euthanasia, from which 42–80% (median 65.3%) was liquid sucrose (Figure 1c,d). Solid food and liquid sucrose intake were significantly reduced in RYGB and consequently PF groups post-surgery compared to the AdLib group (Figure 1c,d). PF animals were fed 100–106 kcal/day, of which 62–63% (median 62.7%) corresponded to the liquid sucrose diet. AdLib animals consumed 126–177 kcal/day, 62–73% (median 69.3%) derived from a liquid sucrose diet. The total energy consumed by AdLib animals was significantly higher than by RYGB and PF, while the percentage of median energy consumption via liquid sucrose was not significantly different between the RYGB and sham.

### 2.2. RYGB Surgery Leads to Reduced Relative Abundance of Firmicutes and Increased Bacteroidota and Proteobacteria Species

**Figure 2:**
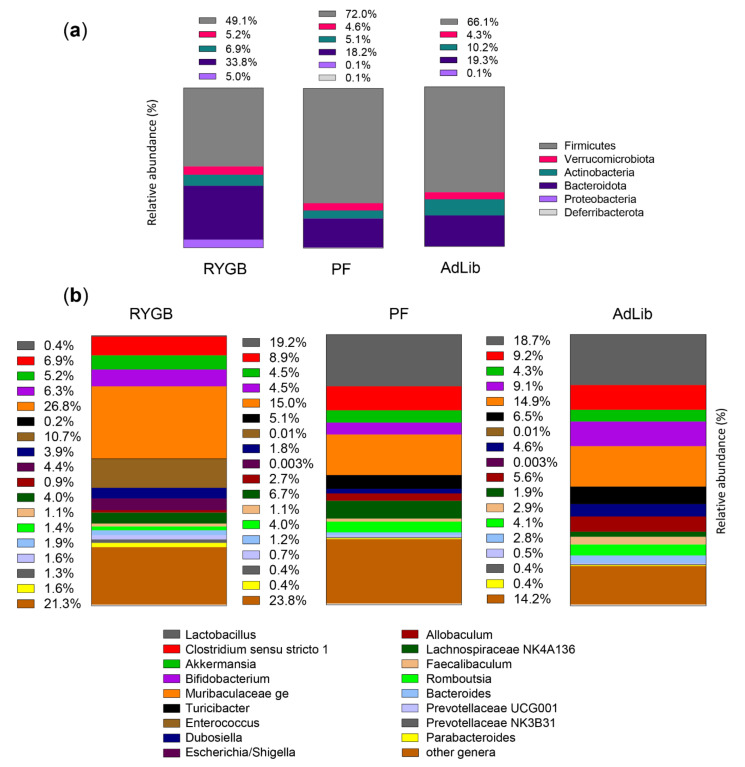
Gut microbiota composition. Bar graph representation of the most abundant taxa at the phylum (**a**) and genus (**b**) levels, illustrating a different microbiota composition between the groups with the RYGB animals displaying a higher abundance of Bacteroidota (particularly *Muribaculaceae*) and Proteobacteria (assigned to the family *Enterobacteriaceae*, genera *Escherichia*/*Shigella*), and a lower abundance of Firmicutes, even if more *Enterococcus* were found in this group, than in the Sham (PF and AdLib) animals, which had more *Lactobacillus* in their microbiota. Data from the three feces collection times are pooled. Mean relative abundance as % of the whole community is shown.

RYGB animals displayed a higher abundance of Bacteroidota (particularly *Muribaculaceae*) and Proteobacteria (Enterobacteriaceae, genera *Escherichia*/*Shigella*) and a lower abundance of Firmicutes compared to sham (Figure 2a,b). RYGB had increased Enterococcus species compared to the sham animals with more *Lactobacillus* species in their microbiota.

Considering feces microbiome at four and eight weeks after surgery and on the day of euthanasia together, a significant difference in alpha-diversity between the different groups (*p* < 0.01 for all three diversity types) was observed with AdLib animals showing the lowest diversity (Supplementary Figure S1). There were no significant changes in alpha-diversity over time within each condition, indicating relatively stable bacterial communities over the study period (data not shown).

Principal coordinate analysis based on the Bray–Curtis distance, Jenson–Shannon, and weighted UniFrac of all samples showed a clear separation between RYGB and sham animals, of which the two sham groups (AdLib and PF) had a visible overlap (Supplementary Figure S1b).

The permutational multivariate analysis of variance (PERMANOVA) with the Bray–Curtis distance showed a highly significant effect of surgery (*p* = 0.001), time (*p* = 0.003), without interaction between surgery and time effects (*p* = 0.3). The linear discriminant analysis effect size (LefSe) led to 16 significant taxa discriminating between groups: 5 for the PF animals, 5 for the AdLib, and 6 for the RYGB rats (Supplementary Figure S1c), represented in a cladogram (Supplementary Figure S2d). As shown above, *Enterococcaceae* was a significant marker of the RYGB animals. A potential marker for the PF animals was *Lactobacillus* and *Bifidobacterium* species up to their phylum of Actinobacteria for the AdLib animals, all with a linear discriminant analysis (LDA) score > 4.

### 2.3. Fecal Metabolomics Show Altered Metabolite Production Post-Surgery

**Figure 3:**
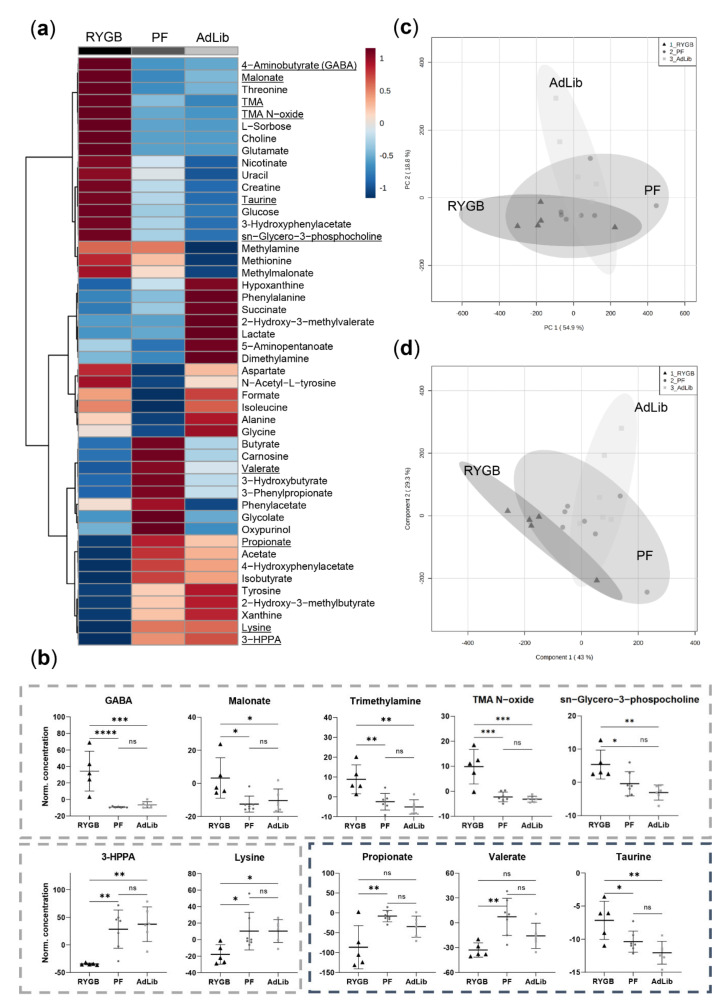
Feces metabolomics analysis eight weeks post-surgery. (**a**) Averaged heat map representing all the quantified metabolites with their relatively elevated (red) or lowered (blue) concentration differences between the groups. Metabolites significantly different between groups (one-way ANOVA) are underlined and represented as box plots (**b**): upregulated 4-aminobutyrate (GABA), malonate, trimethylamine (TMA), TMA N-oxide and sn-glycero-3-phosphocholine, and downregulated 3-hydroxyphenylpropionate (3-HPPA) and lysine in the RYGB animal group (grey dashed border signifies the metabolites that exhibited the same pattern already at four weeks post-surgery). Further, eight weeks post-surgery propionate and valerate were significantly downregulated, and taurine was upregulated in RYGB. Individual samples are represented as RYGB (*n* = 5) black triangles, PF (*n* = 8) dark grey dots, AdLib (*n* = 6) light grey squares with mean and standard deviation. *p*-values: **** < 0.0001, *** < 0.001, ** < 0.01, * < 0.05. (**c**) Principal component analysis (PCA) and (**d**) partial least squares discriminant analysis (PLSDA) regression model illustrate an overall sample cluster overlap. 95% confidence interval is represented as grey clouds.

Eight weeks post-surgery, we identified significantly downregulated 3-hydroxyphenylpropionate (3-HPPA) and lysine in RYGB compared to sham (PF and AdLib) in animal feces (Figure 3a,b). On the contrary, GABA, malonate, TMA, TMAO, and sn-glycero-3-phosphocholine (GPC) concentrations were significantly upregulated in RYGB compared to PF and AdLib (Figure 3b). These metabolite changes were already observed four weeks post-surgery (Supplementary Figure S2b).

Moreover, significantly lower propionate, valerate, and upregulated taurine concentrations were quantified in RYGB compared to sham groups eight weeks post-surgery (Figure 3b). All three group clusters, however, did not completely separate in principal component analysis (PCA), and partial least squares discriminant analysis (PLSDA) illustrated a general overlap (Figure 3c,d).

At the end of the study period, RYGB compared to PF, but not to AdLib, exhibited lowered short-chain fatty acids (SCFA) butyrate, acetate, and formate in feces from the colon (Supplementary Figure S3a,b). We further observed an increased sample scattering in the RYGB group in PCA and PLSDA (Supplementary Figure S3c,d).

### 2.4. RYGB Surgery-Altered Microbiota Composition Correlates with the Feces Metabolite Changes

**Figure 4:**
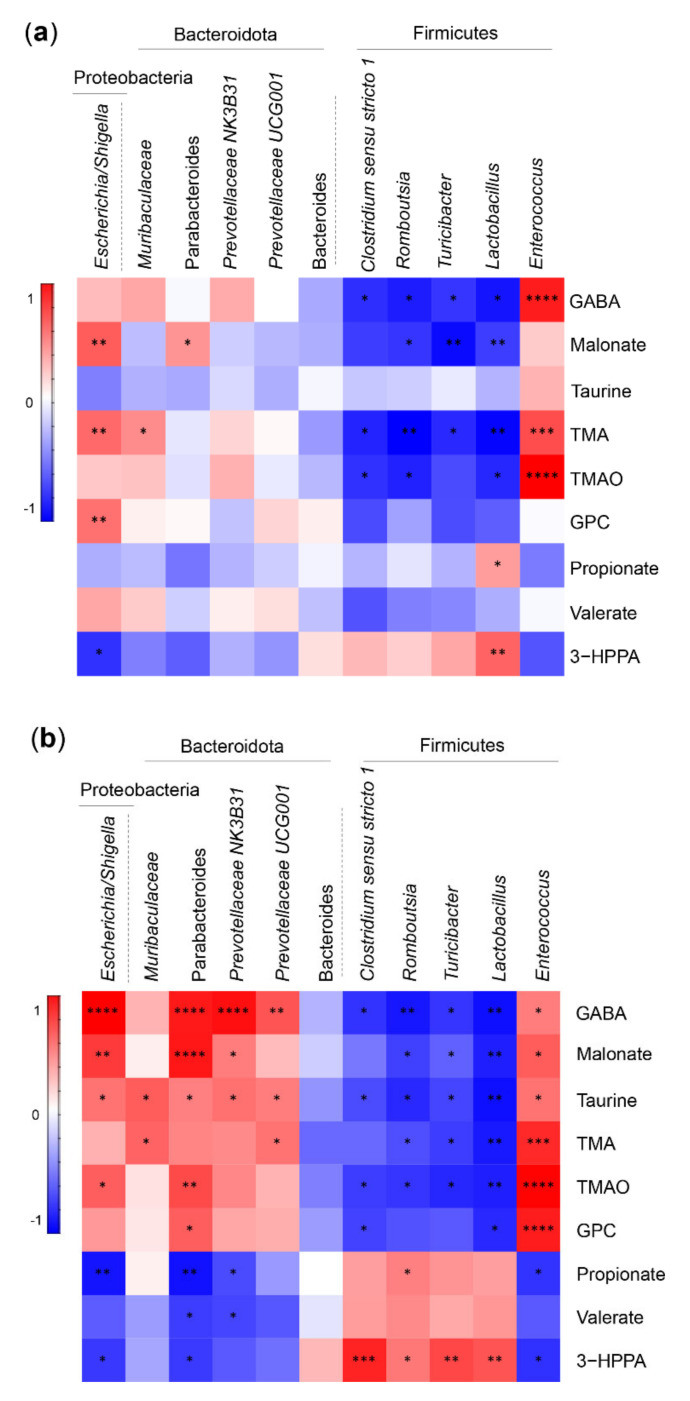
Fecal microbiota and metabolomics correlation analysis. (**a**) Four weeks and (**b**) eight weeks post-surgery. The color scheme illustrates the correlation values scaled between −1 and 1. The red color indicates a positive correlation, blue—negative correlation. Statistical significance of these correlations illustrated as *p*-values: **** < 0.0001, *** < 0.001, ** < 0.01, * < 0.05. Correlation analysis involves all three group values (RYGB, PF, and AdLib pooled), correlation coefficients from the Pearson r distance measure. Abbreviations: GABA—4-aminobutyrate, TMA—trimethylamine, TMAO—trimethylamine N-oxide, GPC—sn-glycero-3-phosphocholine, and 3-HPPA—3-hydroxyphenyl propionate.

Next, we performed correlation analyses between the gastrointestinal microbiota and their metabolites in feces four and eight weeks after the surgery (Figure 4) and at the end of the study (Supplementary Figure S4). This analysis focused on the most significant feature differences between RYGB and both sham groups pooled. The main correlation patterns were present already four weeks post-surgery (Figure 4a, and remained at the eight weeks post-surgery (Figure 4b: GABA, malonate, taurine, TMA, TMAO, and GPC concentrations positively correlated with the relative abundance of the Proteobacteria *Escherichia*/*Shigella* and the Bacteroidota *Muribaculaceae*, Parabacteroides, *Prevotellaceae NK3B3*, *Prevotellaceae UCG001* as well as the Firmicutes *Enterococcus* species while propionate, valerate, and 3-hydroxyphenylpropionate (3-HPPA) negatively correlated to the abundance of these taxa. Furthermore, negative correlations were observed between GABA, malonate, taurine, TMA, TMAO, and GPC and the Firmicutes *Clostridium sensu stricto 1*, *Romboutsia*, *Turicibacter*, *Lactobacillus*, and the Bacteroidota *Bacteroides* species (Figure 4 and Figure S4).

At the end of the study, the concentrations of fecal metabolites isoleucine and 3-hydroxybutyrate positively correlated with the relative abundance of some Bacteroidota, Parabacteroides, *Prevotellaceae NK3B31*, *Prevotellaceae UCG001*, and also with the abundance of the genera *Enterococcus* and *Escherichia/Shigella* (Supplementary Figure S4). Acetate, propionate, butyrate, and valerate concentrations correlated negatively with the abundance of the same species. Isoleucine showed a negative correlation with *Lactobacillus* species.

### 2.5. RYGB Surgery Changes the Branched-Chain Amino Acid, Serine, and Glycine Metabolism in Plasma

**Figure 5:**
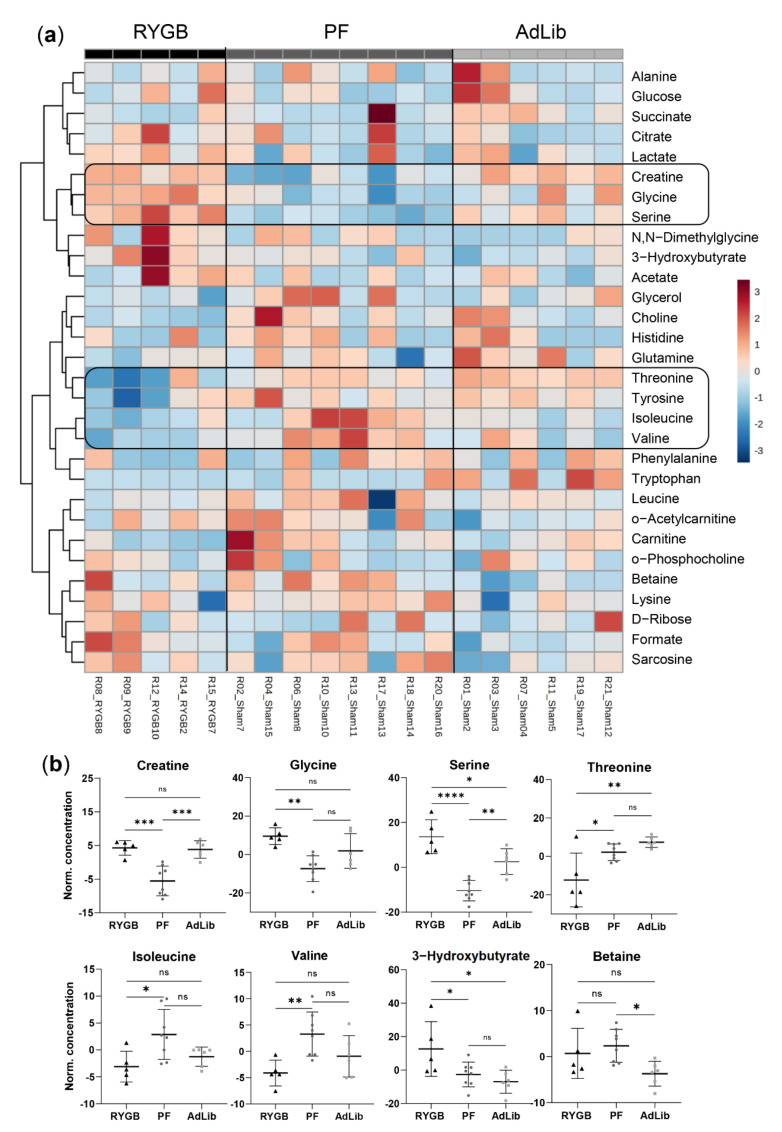
Plasma metabolite quantification analysis at the day of euthanasia. (**a**) Heat map illustration of all the metabolites profiled in all plasma samples, with unsupervised clustering towards similar metabolic patterns. (**b**) Individual box plots of one-way ANOVA significant metabolites: RYGB (*n* = 5) black triangles, PF (*n* = 8) dark grey dots, AdLib (*n* = 6) light grey squares, mean and standard deviation, *p*-values: **** < 0.0001, *** < 0.001, ** < 0.01, * < 0.05.

Plasma metabolomics revealed different metabolic patterns between the RYGB, AdLib, and the PF animal groups (Figure 5a and Figure S5a). Serine and glycine were increased in the RYGB group compared to the PF animals, but not AdLib (Figure 5b and Figure S5b). Creatine levels in RYGB rat plasma were elevated compared to sham animals. Moreover, isoleucine and valine were downregulated in RYGB. These metabolites are connected via glycine, serine & threonine, and glyoxylate & dicarboxylate metabolic pathways. Furthermore, downregulated threonine, valine, and isoleucine further indicated an affected glycine, serine & threonine metabolism pathway and valine, leucine & isoleucine (BCAA) biosynthesis pathway. We also saw downregulated betaine in the AdLib group compared to PF and RYGB. All three groups had many unchanged metabolite concentrations leading to a general group confidence region overlap in PCA (Supplementary Figure S5c). Several metabolites had highly variable concentrations within a group. PLSDA regression model revealed an increased sample scattering in RYGB compared to sham groups (Supplementary Figure S5d).

### 2.6. RYGB Animals Have Reduced C-Reactive and Increased Lipopolysaccharide-Binding Proteins in Plasma

**Figure 6:**
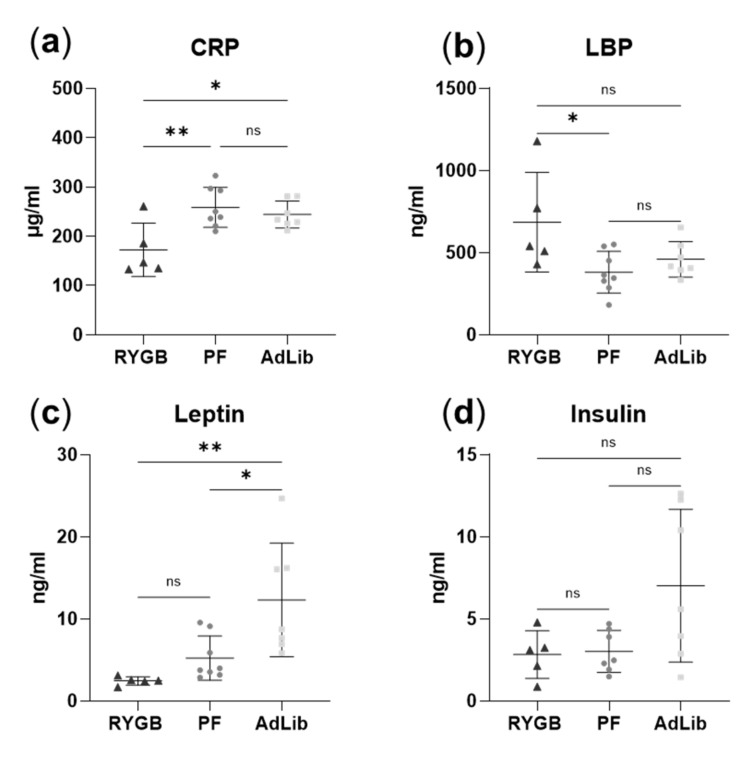
Immune and hormonal parameter profiling. Box plots visualizing the downregulated C-reactive protein (CRP) (**a**) and upregulated lipopolysaccharide-binding protein (LBP) (**b**) in RYGB compared to PF. Leptin was downregulated both in RYGB and PF animals compared to AdLib (**c**). No significant changes in insulin concentration were detected between groups (**d**). One-way ANOVA statistical significance, individual values illustrated for RYGB as black triangles (*n* = 5), PF dark grey dots (*n* = 8), AdLib light grey squares (*n* = 7), with mean and standard deviation, *p*-values: ** < 0.01, * < 0.05.

We quantified the concentrations of the acute-phase protein C-reactive protein (CRP), the lipopolysaccharide-binding protein (LBP), the appetite-reducing hormone leptin, and different cytokines in plasma from the three animal groups (Figure 6 and Figure S6). CRP plasma levels were significantly reduced in the RYGB group compared to the PF and the AdLib group (Figure 6a). On the other hand, LBP was significantly increased in RYGB plasma compared to PF (Figure 6b). Leptin levels were also significantly increased in the AdLib compared to the PF and RYGB (Figure 6c). Insulin concentrations were constant across the groups, with a large variation observed in the AdLib group (Figure 6d). Similarly, no differences were detected for monocyte chemoattractant protein (MCP) 1, interleukin (IL) 6, IL-10 (Supplementary Figure S6a). Chemokine ligand (CXCL) 2, IL-1α, tumor necrosis factor (TNF)-α, and interferon (IFN)-γ were below the limit of detection (Supplementary Figure S6b).

### 2.7. The Hepatic Fatty Acid Profile Changes with Liquid Sucrose Diet after RYGB Surgery

**Figure 7:**
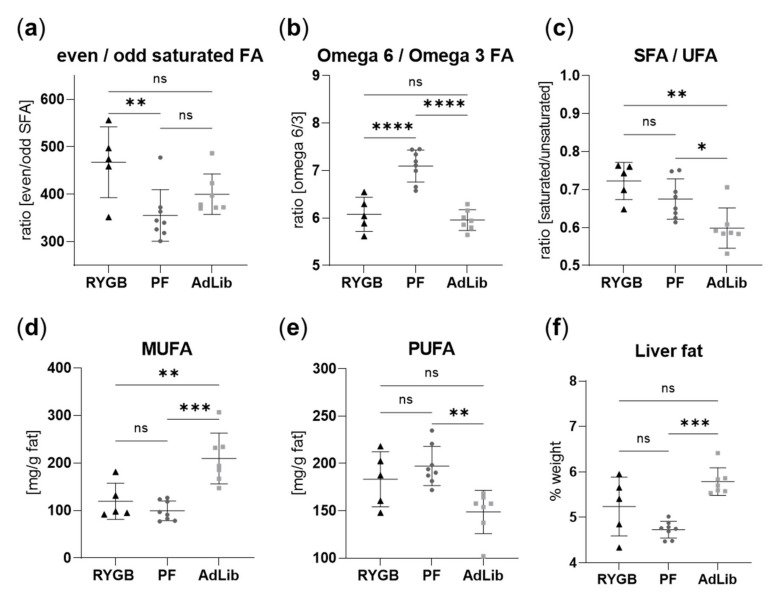
Hepatic fatty acid composition in RYGB and sham-operated animal groups. (**a**) Even to odd chain fatty acid (FA) ratio (*w*/*w*), (**b**) omega 6 to omega 3 FA ratio (*w*/*w*), (**c**) saturated (SFA) to unsaturated FA (UFA) ratio (*w*/*w*), (**d**) mono-unsaturated fatty acid (MUFA) concentration (mg/g fat), (**e**) poly-unsaturated fatty acid (PUFA) concentration (mg/g fat), (**f**) liver fat (% weight of liver). One-way ANOVA statistical significance, individual values illustrated for RYGB as black triangles (*n* = 5), PF dark grey dots (*n* = 8), AdLib light grey squares (*n* = 7), with mean and standard deviation, *p*-values: **** < 0.0001, *** < 0.001, ** < 0.01, * < 0.05.

As both diet and microbiota can influence the liver lipid metabolism, we quantified the lipid fraction in the liver and its fatty acid (FA) composition. The overall lipid profile was influenced by both the surgery and food intake. Even/odd chain saturated FA ratio (C15:0, pentadecylic acid; C17:0, margaric acid) was significantly higher in hepatic fat of the RYGB animals compared to the PF group (Figure 7a and Figure S7a,b). Moreover, the hepatic omega 6 to omega 3 FA ratio was significantly higher in the PF animals compared to AdLib and RYGB (Figure 7b). Omega 6/3 ratio of the RYGB group was not significantly different compared to the AdLib group even though C22:5(4,7,10,13,16) (docosapentaenoic acid, (PUFA, omega-6)) was increased in RYGB compared to the sham groups (Supplementary Figure S7c).

Independent from the surgery-specific observations, food intake restriction and liquid sucrose diet changed FA liver profiles. The most notable differences in the AdLib group were related to the FA saturation compared to RYGB and PF. The saturated/unsaturated FA ratio was higher in the RYGB and PF when the food was reduced, compared to AdLib (Figure 7c), mainly due to low MUFA concentrations (Figure 7d). In contrast, less poly-unsaturated FA was detected in AdLib animal liver compared to RYGB and PF (Figure 7e).

Corresponding differences in the AdLib group were also clearly visible in the plots of several single FA. Upregulated C16:1(9) (palmitoleic acid, SFA), C18:1(9) (oleic acid, MUFA)), lowered C18:0 (stearic acid, SFA) and lowered C20:4(5,8,11,14) (arachidonic acid, PUFA, omega-6) and C22:4(7,10,13,16) (docosatetraenoic acid, PUFA, omega-6) were quantified in AdLib rats to RYGB and PF (Supplementary Figure S7d–h). Consequently, liver fat content (%) was higher in the AdLib group compared to PF yet not statistically significant when compared to RYGB (Figure 7f).

Finally, we observed a higher desaturase activity in the AdLib group compared to PF. A lower elongase activity was quantified in the AdLib rats compared to PF and RYGB (Figure 7g,h and Figure S7f,l,m). Besides the fatty acid distribution in the liver, we also quantified the expression of key genes of hepatic lipid metabolism. Only fatty acid-binding protein 1 (FABP1) had an altered expression between groups in our investigated cohort, higher in the AdLib animals than in the PF (Supplementary Figure S7k). Total energy intake correlated positively to MUFA but negatively to the C24:0 concentration (Supplementary Figure S7n,o). Expression of fatty acid synthase (FAS) positively correlated to the percentage of energy obtained from the liquid sucrose solution (Supplementary Figure S7p).

### 2.8. The Correlation of Metabolomics Data with Plasma Metabolomics, Immune, Hormonal, and Liver Parameters Indicates Surgery-Specific and Food-Related Patterns

**Figure 8:**
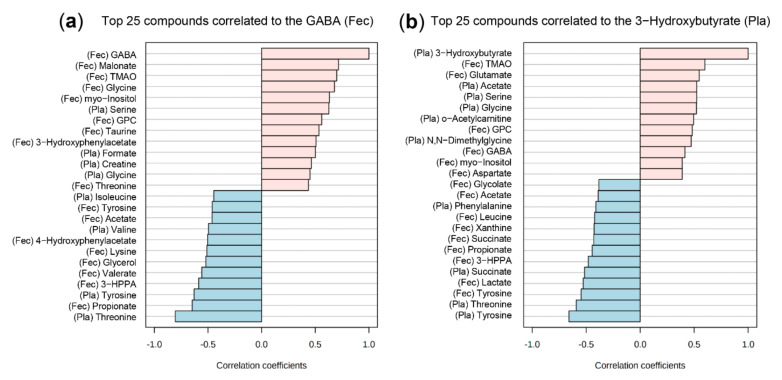
Correlation analysis between the plasma and feces metabolites. Top 25 compounds correlated with (**a**) GABA and (**b**) 3-hydroxybutyrate. Correlation coefficients are based on Pearson r distance measure. Abbreviations: Pla—plasma metabolite, Fec—fecal metabolite, GABA—4-aminobutyrate GPC—*sn*-glycero-3-phosphocholine, 3-HPPA—3-hydroxyphenylpropionate, TMAO—trimethylamine N-oxide.

We correlated plasma and fecal metabolite concentration patterns to investigate further the systemic effects of the RYGB surgery (Figure 8). Fecal GABA had the highest positive correlation with fecal malonate and TMAO, while it had negative correlations with plasma threonine, tyrosine, fecal propionate, and 3-HPPA (Figure 8a). Plasma 3-hydroxybutyrate correlated with fecal TMAO and glutamate and had a negative correlation with plasma tyrosine and threonine, as well as fecal tyrosine, lactate, 3-HPPA, propionate, and acetate (Figure 8b).

For a cross-platform comparison, we further correlated the significantly differing plasma and feces metabolites with significantly changed immune and hormonal parameters and liver FA concentration changes. Of note, these comparisons correlate between the most prominent feature changes, considering the RYGB group compared to sham. First, we identified a prominent group of features similar between PF and AdLib but significantly different to the RYGB group (Supplementary Figure S8a). Fecal taurine and TMA positively correlated to LBP while fecal TMAO and GABA positively correlated to C22:5(4,7,10,13,16) (docosapentaenoic acid (osbond acid), PUFA, omega-6), and C24:1(15) (nervonic acid, MUFA). C22:5(4,7,10,13,16) also positively correlated to plasma 3-hydroxybutyrate and negatively correlated to plasma threonine. CRP had positive correlations with fecal 3-HPPA, plasma threonine, and a negative correlation with fecal GPC, GABA, and TMAO. Second, we identified similar features between the RYGB and PF groups that were significantly different from the AdLib group (Supplementary Figure S8b). Plasma leptin negatively correlated to C22:4(7,10,13,16) (docosatetraenoic acid, PUFA, omega-6) while it had positive correlations to the sum of MUFA, C16:1(9) (palmitoleic acid, MUFA), and C14:0 (myristic acid, SFA). FABP1 positively correlated to C15:0 (pentadecylic acid, SFA), C16:1(9) (palmitoleic acid, MUFA), and liver fat % content.

### 2.9. Brain Imaging Reveals Increased Neuronal Activity after RYGB Surgery

**Figure 9:**
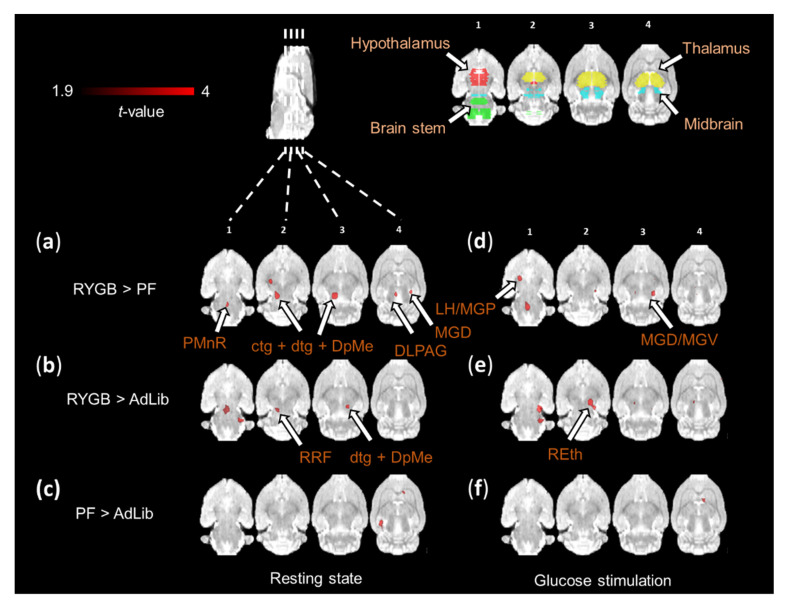
Functional PET brain activation maps at resting state and after glucose stimulation. Coronal brain sections show increase in relative [^18^F]FDG uptake at resting state and after glucose stimulation. Comparison of (**a**,**d**) RYGB > PF rats, (**b**,**e**) RYGB > AdLib and (**c**,**f**) PF > AdLib at a significance level of *p* ≤ 0.01. Brain region color code: red—hypothalamus, green—brain stem, yellow—thalamus, blue—midbrain. RYGB (*n* = 5), PF (*n* = 5) AdLib (*n* = 6). Abbreviations: PMnR—paramedian raphe nucleus, ctg—central tegmental tract, dtg—dorsal tegmental bundle, DpMe—deep mesencephalic nucleus, MGD—medial geniculate nucleus dorsal part, LH—lateral hypothalamus, MGP—medial globus pallidus, MGV—medial geniculate nucleus ventral part, RRF—retrorubral field, REth—retroethmoid nucleus.

Neuronal activity was investigated using [^18^F]fluorodeoxyglucose (FDG) PET at resting state and after a glucose stimulus. When comparing RYGB to PF rats, [^18^F]FDG tracer uptake at resting state was increased in the brain stem and midbrain areas, including the central tegmental tract (ctg), dorsal tegmental bundle (dtg), deep mesencephalic nucleus (DpMe), dorsolateral periaqueductal grey (DLPAG), and medial geniculate nucleus dorsal (MGD) (Figure 9a). Similarly, neuronal activation at the resting state in RYGB was increased in the brain stem and midbrain areas, such as retrorubral field (RRF), dtg, and DpMe compared to AdLib (Figure 9b).

Furthermore, increased neuronal activity in response to glucose stimulation was observed in thalamic areas, such as MGD, medial geniculate nucleus ventral (MGV), and the hypothalamic regions, such as the lateral hypothalamus (LH) and medial globus pallidus (MGP) in RYGB animals compared to PF (Figure 9d). The thalamic areas were also activated when comparing RYGB to AdLib (Figure 9e). No specific differences in tracer uptake were observed in the investigated brain regions before or after the glucose stimulation when comparing AdLib to the PF group (Figure 9c,f).

In addition, the effect of glucose stimulus was analyzed on a cellular level by early gene (c-Fos) histochemical staining. Generally, glucose-induced c-Fos cell counts were low, with no group differences observed in the basolateral amygdala (BLA), layer 2 of the cortex (L2), or paraventricular hypothalamic (PVH) regions (Supplementary Figure S9; Supplementary Tables S1–S3).

## 3. Discussion

### 3.1. Liquid Sucrose-Induced Obesity Is Reverted by RYGB Surgery

Our results confirmed the persistent weight reduction in liquid sucrose diet-induced obese rats post-RYGB surgery, similar to other animal studies [20,42] and clinical practice [43]. PF animals had similar body weight to the AdLib group after the sham surgery, suggesting that caloric restriction was not the only driver for the RYGB-induced weight loss. Moreover, caloric restriction efficiently reduced total liver fat content in the PF animals. While the total energy consumption was significantly higher in AdLib compared to RYGB and therefore also PF, the relative percentage of energy consumed via liquid sucrose was not significantly changed between the groups. Therefore, we conclude that the surgery led to a reduced total energy intake in RYGB animals.

### 3.2. Microbiota Perturbations Lead to Upregulated Fecal GABA and Lower Fiber Fermentation

RYGB surgery causes a persistent environmental change of the gut which has been previously associated with a long-lasting impact on the microbiota composition and metabolism [44]. RYGB has also been shown to promote a change in dietary habits and their associated neuronal processes [45]. Bacteroidetes and Proteobacteria such as *Escherichia* have been reported to actively express the genes necessary for producing the neurotransmitter GABA in human stool [46]. Here we quantified a higher fecal concentration of GABA in RYGB rats, correlating with a higher relative abundance of *Escherichia*/*Shigella* and Parabacteroides in these animals. The regulation of neurotransmitters through the microbiota can affect the host through gut-brain communication (reviewed in [47,48]). GABA is a crucial part of the brain’s GABAergic system and the enteric nervous system (ENS), acting as a modulator for the gut signaling processes [49]. It has been reported that GABA can activate gut/intestinal cells and further promote a cascade leading to neuronal cell activation via gut secreted exosomes [50]. GABA receptors also have been shown to influence the liquid secretion processes (GABA-A) and gut motility (GABA-B) [51]. Increased GABA concentrations in feces correlate to the liquid state of the feces and suggest a higher defecation rate in RYGB compared to sham groups.

Further, we quantified a reduced relative abundance of Firmicutes in RYGB compared to AdLib and PF feces, particularly *Lactobacillus*, *Turicibacter,* and *Romboutsia*, which are known to produce SCFA via fiber fermentation [38,52]. Lower levels of fecal SCFA in RYGB and AdLib compared to PF may result from different nutrient availability in the colon. In RYGB, this is a potential side effect of surgery and consequent malabsorption as the macronutrients can escape directly from the small intestine into the large intestine. While in AdLib, similarly lower SCFA was quantified due to the larger amount of food ingested, and thus more macronutrients entered the fermentative section of the gut. As the substrate is different, the readily available macronutrients such as protein and simple carbohydrates might be fermented first and fast by the microbiota, giving the SCFA more time for entering enterocytes and the bloodstream via diffusion, resulting in lower fiber fermentation and therefore less SCFA in RYGB and AdLib feces compared to PF.

Moreover, the higher relative abundance of Bacteroidetes, particularly *Muribaculaceae* species, which are versatile carbohydrate degraders [53], could indicate a shift from fiber fermentation to host-derived carbohydrate utilization in the microbiome of RYGB animals [38,54].

Increased fecal taurine in RYGB has been previously reported in the context of the intestinal NOD-like receptor family pyrin domain containing 6 (NLRP6) inflammasome expression repair leading to gut immune homeostasis [55] and supports our findings of reduced inflammation in RYGB as reflected by decreased CRP plasma levels.

TMA and TMAO were elevated in the feces of RYGB rats and are usually produced from dietary choline and carnitine by gut bacterial enzymes (choline TMA lyase; carnitine oxidoreductase) [56,57]. In our dataset, plasma TMA and TMAO had a significant positive correlation to the relative abundance of *Enterococcus,* most prominent at four and eight weeks post-surgery. *Enterococcus* species have been reported to enable plasma TMAO degradation ex vivo [58], suggesting that bacterial species could be activated with the purpose of TMA and TMAO degradation. TMA and TMAO have been related to increased risk of cardiovascular disease [59,60,61]. However, we also observed in the RYGB rats a significant decline of TMAO concentrations in feces from four to eight weeks. Furthermore, in feces samples on the day of euthanasia, TMAO was no longer significantly increased in RYGB compared to sham, which indicates a temporary microbiota shift after the surgical intervention that could be resolved during the recovery time after surgery.

### 3.3. RYGB Surgery Leads to Lowered Plasma BCAA and Increased Glycine

Plasma metabolite profiling confirmed that RYGB surgery alters the valine, leucine, and isoleucine BCAA biosynthesis pathway [62]. BCAA upregulation has been linked to obesity and diabetes [63] and interconnected with glycine downregulation [64]. Reduced solid food intake resulted in less available protein in RYGB and PF. However, quantified BCAA decrease could indicate malabsorption in RYGB.

Furthermore, we identified glycine, serine, and threonine, and glyoxylate and dicarboxylate metabolic pathway upregulation after RYGB surgery. Metabolites such as serine and glycine are known substrates for the folate and methionine cycle [65]. These further participate in one-carbon metabolism, which is crucial for appropriate nucleotide and cofactor synthesis. Obesity and type 2 diabetes have been previously associated with depleted circulating glycine and the potential necessity of therapeutic glycine supplementation [66]. We conclude that RYGB surgery leads to improved plasma metabolite patterns and, therefore, a potentially reduced risk of type 2 diabetes.

### 3.4. RYGB Surgery Results in Elevated LBP Not as a Result of Inflammation but Possibly from Increased Lipolysis as Seen by Plasma Ketone Body Levels

Although increased LBP in plasma is a known marker for inflammation [67], we did not observe any other signs for an upregulated inflammatory response in the RYGB group compared to the sham. Indeed, plasma CRP levels were decreased in RYGB compared to sham groups, which is in good agreement with previous research [68]. Furthermore, our reported microbial changes of increased gram-negative Bacteroidetes and Proteobacteria, which are known to express the antigen LPS on their cell surface [69], and decreased gram-positive Firmicutes, could lead to a higher LPS concentration within the RYGB gut compared to sham. Increased hepatic even/odd chain fatty acid ratio and reduced leptin levels in RYGB support the hypothesis that RYGB leads to upregulated lipolysis as reflected by increased levels of the ketone body 3-hydroxybutyrate in RYGB animal plasma. 3-Hydroxybutyrate is produced by the liver in lipolysis and serves as the primary energy substrate via beta-oxidation and acetyl-CoA for the TCA cycle when glucose is depleted [70]. During lipolysis, long-chain fatty acids can be transported only within chylomicrons, which are therefore highly upregulated [71]. However, as LPS has a high affinity for chylomicrons, we conclude that chylomicron formation due to increased lipolysis promotes intestinal LPS absorption, as previously described in gut mucosa [68]. We thus hypothesize that due to reduced solid food intake and malabsorption, de novo lipogenesis is enhanced in RYGB animal livers from excess sucrose, particularly from the fructose moiety. However, as too many free fatty acids can be toxic, the body might try to regulate this by simultaneously increasing lipolysis, as seen by elevated 3-hydroxybutyrate levels in RYGB plasma. Moreover, increased lipolysis might contribute to the weight loss in the RYGB group that could not be attributed solely to reduced food intake in PF.

Finally, the decrease of the ketogenic amino acid lysine in RYGB feces and increased 3-hydroxybutyrate in RYGB plasma indicates that lysine could be transformed into 3-hydroxybutyrate. In this context, it is important to note that increased 3-hydroxybutyrate has also been found in humans after one-anastomosis gastric bypass surgery [72].

### 3.5. The Even/Odd Saturated FA Ratio Is Increased after RYGB Surgery

The RYGB hepatic FA profiles differed from the PF and AdLib animals when considering the hepatic even/odd FA ratio, which was higher in RYGB. Indeed, RYGB animals showed a lower concentration of margaric acid (C17:0) than sham animals, which could be synthesized in the liver from the gut-derived propionate, a metabolite found in reduced concentrations in the RYGB feces. It is well recognized that gut microbiota influences host lipid metabolism [73]. Kindt et al. showed that microbiota-derived dietary fiber acetate leads to the synthesis of FA in the liver, particularly palmitic acid (C16:0) and stearic acid (C18:0). Moreover, fructose triggers de novo lipogenesis in the liver through two mechanisms, both involving its transformation into acetate by the microbiota [73]. Moreover, the liver can use gut-derived propionate to synthesize odd chain FA [74]. As the odd chain FA increases the membrane fluidity and is linked to a lower risk for type 2 diabetes and could help against Alzheimer’s disease and cancer [75,76,77], our observations suggest an unfavorable effect on RYGB associated with high sucrose consumption on the liver FA profile. Kindt et al. showed that changes in FA profiles in the liver were also found in plasma FA profiles and might, therefore, have a systemic effect. It has also been shown that the circulating long-chain FA can be sensed in the hypothalamus, where they regulate glucose homeostasis [78]. Nevertheless, we did not find a direct gut-brain link through our investigation.

### 3.6. RYGB Surgery Leads to Altered Neuronal Activity in the Rat Brain

We investigated the effect of an RYGB surgery on brain activity by [^18^F]FDG-PET. We observed several activated brain areas in rats that underwent RYGB surgery as a possible result of the ketogenic state where 3-hydroxybutyrate replaced glucose as the primary cellular energy source. The highest neuronal activation was observed in RYGB compared to sham rats in the brain stem, midbrain, thalamus, and hypothalamus areas. The midbrain regions of ctg and DpMe are GABAergic cell-rich regions with a high potential for GABAergic signaling processes [79]. They are involved in an exaggerated activation of homeostatic feeding circuits and display enhanced brain serotonergic signaling after RYGB surgery [80].

We also quantified increased 3-HPPA, which is formed through the fermentation of tyrosine by *Clostridium*, *Escherichia*, and *Eubacteria* species or via polyphenol metabolism [54]. 3-HPPA has been reported to be able to cross the blood-brain barrier, further serving as a competitive inhibitor for dopamine synthesis [81]. As we saw a neuronal activation in the hypothalamus and midbrain (ctg, dtg), regions where dopamine is produced, a further investigation would be necessary to confirm this connection. Although a limitation of this study was the relatively low animal number per group, our data shows that the RYGB surgery results in increased neuronal activity in the RYGB animals compared to controls.

### 3.7. Key Findings and Future Implications

In conclusion, RYGB surgery successfully reversed the weight gain induced by the liquid sucrose diet. In the gut, RYGB rats showed increased Bacteriodota, Probacteria, and decreased Firmicutes. Microbiota changes led to notably increased GABA production in the gut, which we further quantified in feces, possibly influencing downstream metabolite and cytokine profiles as well as signaling in GABAergic regions of the ENS and CNS. Lowered plasma BCAA and increased glycine in RYGB suggested a lowered risk of obesity. As we opted to induce obesity using the liquid sucrose diet, most of the changes in the liver fatty acid profile were related to the diet and caloric restriction effects. We also observed increased brain neuronal activity after the surgery, yet further confirmatory investigations are needed to truly underpin the molecular mechanisms regulating appetite, metabolism, and gut-brain signaling.

## 4. Materials and Methods

### 4.1. Animals

Male Lewis rats (*n* = 27, approximately 400 g, (Charles River, Sulzfeld, Germany) were single-housed in IVC cages (1500U) enriched with nesting material and kept on a 12-h day-night cycle at a room temperature of 22 °C and 40–60% humidity. All experimental procedures were performed in accordance with the European Union Directive 2010/63/EU on the protection of animals used for scientific purposes and were approved by the Regierungspraesidium Tuebingen (Tuebingen, Germany regional authorities approval, permit number MPV 3/16, issued 13 May 2019).

### 4.2. Experimental Design and Surgical Technique

The experimental design is shown in Figure 1a. Rats (*n* = 27) were randomized in cages, housed in groups of four, and had ad libitum access to phytoestrogen-reduced chow (Sniff, Soest, Germany) and a water bottle containing 30% sucrose (Sigma Aldrich Chemie, Taufkirchen, Germany) in water (liquid sucrose) for eight weeks. Subsequently, rats were randomly assigned to undergo RYGB (*n* = 10) or sham surgery (*n* = 17). The details of the RYGB surgery have been described previously [82]. Rats were fasted overnight and were treated with antibiotics (Baytril, 5.7 mg/kg, s.c., Bayer, Leverkusen, Germany) and analgesics (Carprofen, 5 mg/kg, s.c., Bayer, Leverkusen, Germany) prior to anesthesia. Anesthesia was induced in an induction chamber with 4–5% isofluorane (CP-Pharma Handelsgesellschaft mbH, Burgdorf, Germany). When surgical tolerance was reached, anesthesia was maintained with 1–3% isofluorane. The surgical areas were cleaned with Braunol (B Braun SE, Hessen, Germany), and all rats received eye ointment to protect the cornea. Rats were kept on heating pads to avoid hypothermia. Following a midline laparotomy, the RYGB surgical intervention consisted of a transection of the stomach, leaving only a small proximal pouch, a transection of the small bowel, and reanastomosis creating an alimentary (or Roux) limb of approximately 50 cm, a biliopancreatic limb, and a common channel. The esophagogastric junction was anastomosed to a loop of jejunum 8–10 cm distal to the ligament of Treitz in an end-to-side fashion. A 7-mm side-to-side small bowel anastomosis was performed between the biliopancreatic and the alimentary limbs to create a common channel of approximately 25 cm. Anastomoses were performed using prolene 7/0 and the gastric remnant was closed with prolene 5/0. The sham procedure consisted of a laparotomy, a 7-mm gastrotomy on the anterior wall of the stomach, and resuturing of the gastrotomy with 5/0 prolene. At the end of all the procedures, 5 mL of warm 0.9% saline was given i.p. to compensate for fluid loss before the closure of the abdominal wall and skin with vicryl 4/0. Rats were maintained in a warm environment until they were fully awake and mobile. Rats received antibiotics and analgesics (as described in the pre-operative preparation) on postoperative days 1–4, once a day, and single-housed to avoid any additional risk of wounds. Four RYGB rats died shortly after the surgery. During the first week after surgery, wet food was provided in the cages for recovery, and no sucrose was given to avoid inflammation in RYGB animals. Sucrose intake started again one week after surgery. After nine weeks, the RYGB group (*n* = 6) and sham AdLib group (*n* = 9) continued to have ad libitum access to the diet and liquid sucrose, while the sham pair-fed group (PF; *n* = 8) received water ad libitum and were pair-fed chow and liquid sucrose solution calculated from the food and liquid sucrose consumption of the RYGB group. One RYGB rat (RYGB 6) was euthanized 6 weeks after RYGB surgery as weight loss was more than 20% of the average body weight and had reached termination criteria. One AdLib rat (Sham 1) was euthanized as a control to collect data and one AdLib rat (Sham 9) died shortly before the FDG-PET measurement. The final animal numbers per group were RYGB *n* = 5, PF *n* = 8, AdLib *n* = 7.

### 4.3. [^18^F]FDG-PET with Glucose Stimulation

At least eight weeks post-surgery, overnight fasted rats were deeply anesthetized with 3% isoflurane evaporated in the air at a flow rate of 0.8 L/min in an induction chamber. The isoflurane was reduced to 2% for maintenance, and a blood sample was collected from the tail vein to determine the blood glucose concentration. Three catheters were placed into the tail vein for anesthesia, glucose stimulation, and tracer injection, and rats were intubated and connected to a small animal ventilator (DC1 73-3629, Harvard Apparatus, Holliston, MA, USA). Breaths per minute were set to 60 with an inspiration duration of 60% of the ventilation cycle. The end-inspiratory pressure was set to approximately 10 cm H_2_O and 500 mL/min flow. During the preparation, animals were warmed by a heating pad. At least 30 min before the start of the PET acquisition, isoflurane anesthesia was replaced by an initial bolus of 16 mg of alpha chloralose, followed by a second bolus containing 5 mg of alpha chloralose and 0.25 mg of pancuronium bromide after five minutes. A constant infusion of alpha chloralose (20 mg/kg/h) (Sigma Aldrich Chemie GmbH, Taufkirchen, Germany) and pancuronium bromide (1 mg/kg/h) (Inresa Arzneimittel GmbH, Freiburg, Germany) was started and maintained for the duration of the imaging experiment.

[^18^F]FDG was obtained from the radiopharmacy (Department of Preclinical Imaging and Radiopharmacy). PET/MRI experiments were performed on a combined PET/MRI (7T) system (ClinScan, Bruker BioSpin MRI GmbH, Ettlingen, Germany) with an in-house-built PET insert [83]. Using a localizer scan, rats were placed in the center of the field of view (FOV) on a water-heated small animal bed (Medres, Cologne, Germany) connected to a feedback temperature control unit (Medres, Cologne, Germany) set to 37 °C. A 72 mm linearly polarized RF coil (Bruker BioSpin MRI GmbH, Ettlingen, Germany) was used for signal excitation and a transceiver coil for the anatomical sequence. At least 30 min after isoflurane was set to 0%, a bolus plus constant [^18^F]FDG (injected activity: 87.5 ± 9.2 MBq) infusion was started (bolus: 166 µL/min for one minute, infusion 8 µL/min remaining time; 155–165 MBq in 1.1 mL). Dynamic PET data were acquired for 70 min and divided into 70-time frames (60 × 60 s; 1 × 30 s). A one-minute glucose stimulation (0.35 g/250 g body weight, 50% Glucose solution B. Braun, Melsungen, Germany) was injected 30 min after the start of the PET acquisition. At the end of the scan, an anatomical T2 image was acquired using a TurboRARE sequence (TE: 67.11 ms, TR: 1800 ms, rare factor: 28, averages: 1, FOV: 40 mm × 32 mm × 32 mm, image dimensions: 160 px × 128 px × 128 px, voxel size: 0.25 mm × 0.25 mm × 0.25 mm).

### 4.4. [^18^F]FDG-PET Data Analysis

All [^18^F]FDG-PET scans were stored as list-mode files and reconstructed dynamically into one-minute time frames using an ordered subset expectation-maximization 2D (OSEM2D) algorithm. Then PET DICOM images were converted into NIfTI files before pre-processing using Statistical Parametric Mapping (SPM12, Wellcome Trust Centre for Neuroimaging, University College London, London, UK) in MATLAB (Mathworks, Natick, MA, USA). First, image realignment was applied to correct the motion artifacts. Then, skull stripping was performed on all PET and anatomical reference images of each rat using binary masks generated in AFNI (Analysis of Functional Neuro Images, Medical College of Wisconsin, Milwaukee, WI, USA). Next, the PET images of each rat were co-registered to their respective anatomical image using SPM12. The anatomical scans were then used to normalize the PET datasets to the Schiffer rat brain atlas [84]. Finally, a 1.5 × 1.5 × 1.5 mm^3^ full-width-half-maximum (FWHM) Gaussian kernel was applied to all PET images for spatial smoothing [85].

Time-activity curves of each region of interest were normalized to the whole-brain radioactivity. Average normalized PET images were generated for all cohorts between 21 and 30 min and 41 and 50 min for voxel-wise comparison of baseline and glucose-stimulated conditions. The generated images were loaded into a second-level SPM two-sample analysis to generate contrasts between RYGB and AdLib, RYGB and PF, and PF and AdLib based on *t*-test statistics. R02 (Sham 7), R17 (Sham 13), R04 (Sham15), and R01 (Sham 02) data were excluded from further analysis due to distorted images because of animal movement during the scan or a paravenous [^18^F]FDG injection.

### 4.5. Tissue and Feces Collection

Rat feces were collected at four and eight weeks post-surgery from the cage. After the PET scan, 1–2 mL blood sample was collected and centrifuged for 5 min at 4 °C 3000 rpm after clotting for 20–30 min at room temperature. Plasma was aliquoted, snap-frozen, and stored at −80 °C until further experiments. Rats were transcardially perfused with 50 mL ice-cold PBS followed by 150 mL of 4% paraformaldehyde (PFA). Rat feces were collected from the colon in cryo-vials and snap-frozen in liquid nitrogen. The brains were surgically extracted, post-fixed in 4% PFA overnight, and transferred to PBS the next day for long-term storage at 4 °C. Liver tissue was collected and snap-frozen in liquid nitrogen and kept at −80 °C for long-term storage.

### 4.6. Immunohistochemistry

Early gene (c-Fos) and NeuN histochemical stainings were performed on free-floating sections. The brains were cut into 60-μm coronal slices using a Microm (HM 650 V vibratome Thermo Fisher Scientific, Dreieich, Germany). Individual sections were collected in wells filled with 0.1 M PBS (pH 7.4). Per animal, three coronal sections at the level −1.8 mm posterior to bregma, according to the coordinates of the atlas of Paxinos and Watson [86], were chosen for analysis based on brain activity seen in the PET data.

Neural activity on a cellular level was revealed using antibodies against the c-Fos protein, an early transcription factor. In addition, NeuN, a marker for neurons, was applied to detect how many c-Fos positive cells were neuronal. Brain sections were washed three times with 0.1 M PBS (pH 7.4) and blocked for 2 h with 10% goat serum (s-1000, Vector Laboratories, Linaris Biological Products, Mannheim, Germany) in 1X PBS. Sections were incubated with c-Fos antibody (1:1000, mouse monoclonal (C-10), sc-271243, Santa Cruz Biotechnology, Dallas, TX, USA) for 72 h on a shaker at 4 °C in the dark. Sections were then washed three times with 0.1 M PBS and incubated with the secondary antibody (1:2000, Alexa Flour 488, goat anti-mouse IgG H + L, ab150117, Abcam, Cambridge, UK) for 2 h at room temperature (RT). Sections were subsequently washed 0.1 M PBS and incubated with anti-NeuN antibody (Anti-NeuN, clone A60, mouse, Alexa Fluor^®^555 conjugated, Agilent, Santa Clara, CA, USA) in blocking solution for 72 h at 4 °C. After rinsing with 0.1 M PBS, sections were finally stained with DAPI (Thermo Fisher Scientific Inc., Dreieich, Germany) (10 min, RT) and mounted on glass slides using Fluoromount-G (H-100, VECTASHIELD^®^ Antifade Mounting Medium, Linaris Biological Products, Mannheim, Germany).

### 4.7. Confocal Imaging

Images were captured using a confocal microscopy system (Confocal LSM 710, Zeiss, Jena, Germany). The images were post-processed using linear brightness and contrast transformations to improve visibility. For figures, images were further processed with CorelPhotoPaintX6 (CorelPhotoPaintX6, Corel Cooperation, Ottawa, ON, Canada). Experimenters blinded to experimental groups used the DAPI channel and anatomical guidelines described by Paxinos & Watson [86] to determine regions of interest (ROIs). The basolateral amygdaloid nucleus (BLA), central amygdaloid nucleus (CeC), dorsal endopiriform nucleus (DEn), paraventricular nucleus of the hypothalamus (PVN), and layer 2 of the cortex (L2) were examined for c-Fos and NeuN positive cells.

Analysis was performed using ImageJ software (National Institutes of Health). The number of cells labeled for c-Fos and NeuN and their co-localization were counted manually on the Fiji software (National Institutes of Health (NIH), Bethesda, MD, USA) platform using ImageJ plugin Cell Counter (NIH, Bethesda, MD, USA). The number of counted cells was averaged across three sections per bregma level for each animal and ROI.

### 4.8. 16S rRNA Gene Sequencing-Based Microbiome Analysis

Genomic DNA was extracted from ~200 mg feces using the Nucleospin Stool Kit according to the manufacturer’s recommendation (Macherey Nagel, Düren, Germany) with 1 mL diluted ST1 buffer (1:1 with water), a bead-beating step of 40 s at 6 m/s on a FastPrep 24 G (MP Bio, Illkirch-Graffenstaden, France) instead of step 2. Amplification of 16S RNA gene was performed using a single PCR approach with dual-index V4-region primers (Biomers, Ulm, Germany) [87,88]. The Phusion Hot Start II DNA polymerase (Thermo Fischer Scientific, Walldorf, Germany) was used for 25 cycles on 1 ng of DNA. According to the manufacturer’s instructions (Mag-Bind RXNPure Plus, Omega, Norcross, GA, USA), PCR products were checked on agarose gel and purified with magnetic beads. Quantification was then performed on a Quantus Fluorometer (Promega, Walldorf, Germany) with the QuantiFluor One dsDNA System (Promega, Walldorf, Germany), according to the manufacturer’s recommendation. Products were then pooled in a library (final concentration 8 pM, with 20% PhiX DNA) and sequenced on a MiSeq (Illumina, San Diego, CA, USA) using a MiSeq Reagent Kit v3 for 2 × 300 cycles as recommended by the manufacturer with additional dual-index sequencing primers [89].

Raw sequences were trimmed and quality filtered before ASVs were defined using the package Dada2 [90]. Phyloseq package was used to determine alpha- and beta-diversity [91]. Adonis from the package vegan was used to perform permanova (https://cran.r-project.org/web/packages/vegan/index.html, accessed on 8 October 2020), and the LefSe Server was used to identify significantly discriminative taxa between treatment groups [92].

Raw sequence data have been deposited in the European Nucleotide Archive ENA under the accession number PRJEB49286 (European Nucleotide Archive (ENA).

The 66 sequenced samples led to 2,618,817 reads in total, corresponding to 1520 amplicon sequencing variants (ASVs). Samples had between 12,704 to 78,082 reads. Removing the negative control (around 700 reads) and mock community samples led to 1461 ASVs in rat samples. All ASVs with less than 10 reads overall were removed, resulting in 1106 ASVs on which further analyses were performed.

### 4.9. ^1^H-NMR Spectroscopy-Based Metabolomics Analysis of Plasma and Feces

45 μL of plasma was mixed with 90 μL of LC-MS grade methanol. 300 mg of rat feces were suspended in 400 µL of methanol and 800 µL of methyl-tert-butyl ether (Sigma Aldrich Chemie, Taufkirchen, Germany). Mixtures were subjected to ultrasound metabolite extraction protocol of 5 min per sample by Covaris E220 ultrasonicator equipped with a water-cooling bath at 8 °C (Covaris, Woburn, MA, USA). Further, 500 µL of ultrapure water was added to the feces sample mixture. Glass tubes were centrifuged at 30,000× *g* for 30 min for two-phase separation. The aqueous phase was transferred to a fresh 1.5 mL Eppendorf cup (Eppendorf, Hamburg, Germany). Plasma extract was also transferred to a 1.5 mL Eppendorf cup and centrifuged at 30,000× *g* for 30 min. The supernatant of each sample was transferred to another clean 1.5 mL Eppendorf and evaporated to dryness overnight. Metabolite pellets were re-suspended in 45 μL 200 mM phosphate (K_2_HPO_4_) buffer in deuterated water (D_2_O) containing 200 μM NaN_3_ (pH 7.4), and containing 1 mM (3-(trimethylsilyl) propionic-2,2,3,3-d_4_ acid sodium salt (TSP) internal standard (Sigma Aldrich Chemie, Taufkirchen, Germany). Suspensions were thoroughly mixed (vortexed) and centrifuged at 30,000× *g* for 10 min. 40 μL of clear supernatant were used to fill 1.7 mm NMR tubes (Bruker BioSpin, Ettlingen, Germany).

NMR measurements were carried out on a Bruker Avance III 14.10 Tesla, 600 MHz for ^1^H with a 1.7 mm triple-resonance room-temperature probe (Bruker BioSpin, Ettlingen, Germany). A short zero-go (zg) measurement was followed by 7-min 1D NOESY and 1-h CPMG (512 scans) experiments. Spectra were pre-processed with Bruker TopSpin 3.6.1 software and later profiled using ChenomX 8.5 Professional NMR Suite. For further analysis, R01 (Sham 02) and R20 (Sham 16) feces samples and R16 (Sham 06) plasma samples were excluded due to a technical issue of poor NMR spectra resolution.

### 4.10. Immune and Hormonal Parameter Profiling

Rat interleukin (IL)-10, IL-1α, interferon-gamma (IFN-γ), and tumor necrosis factor α (TNFα) protein levels were quantitatively measured by cytometric bead array flex sets (CBA) (BD Biosciences, San Diego, CA, USA) according to the manufacturer’s protocol. Rat CXCL2, IL-1β, leptin and C-reactive protein (CRP) (R&D Systems, Minneapolis, MN, USA), IL-6 (BioLegend, London, UK), lipopolysaccharide (LPS) binding protein (LBP) (Hycult Biotech, Uden, The Netherlands), and insulin (Crystal Chem Zaandam, The Netherlands) were quantified by enzyme-linked immunosorbent assay (ELISA) according to the manufacturer’s protocol.

### 4.11. Liver Gene Expression

RNA was extracted from 10–25 mg of the frozen mortar-grounded liver using the RNeasy Plus Mini Kit (Qiagen, Hilden, Germany). Reverse transcription was performed with the GoTaq 2-Step RT-qPCR System according to the manufacturer’s recommendation (Promega, Walldorf, Germany), and qPCR was run on a 480 LightCycler (Roche, Basel, Switzerland) with GoTaq qPCR Master Mix 2x (Promega, Walldorf, Germany) for several lipid metabolism-relevant genes and two housekeeping. As beta-actin appears to be highly variable and HPRT highly constant, we kept only HPRT amplification values for the calculation of delta Ct: gene expression values are given relative to this housekeeping gene. Primer sequences are provided in the following Table 1.

### 4.12. Liver Fatty Acid Composition

Liver fat was extracted starting with 200 mg frozen mortar-grounded material in isopropanol-hexane (2:3 vol. with 0.01% BHT), followed by the addition of sodium sulfate and shaking overnight. After centrifugation, the hexane phase was removed and kept, and the rest was washed with isopropanol-hexane (7:2 vol., 0.01% BHT) by vortexing shortly and centrifuging (2000 rpm for 5 min). The pooled hexane phase was dried under nitrogen gas flow, and residual fat was weighed to determine the percentage of fat in liver tissue. Fatty acids were released and derivatized using acetyl chloride in methanol [93]. The composition of resulting fatty acid methyl esters (FAME) was analyzed by gas chromatography-mass spectrometry (Trace GC 1310 with TSQ Duo, Thermo Fischer Scientific, Waltham, MA, USA) using split injection (1:8) [94]. Briefly, FAME were separated on a 70% cyanopropyl column (TR-FAME, 60 m × 0.25 mm × 0.25 µm, Thermo Fischer Scientific, Waltham, MA, USA) with helium carrier gas (1.2 mL/min) and the following temperature gradient: 50 °C (3 min)/50 > 140 °C (15 °C/min)/140 > 220 °C (3 °C/min)/220 > 250 °C (15 °C/min). Individual FAME were identified and quantified using EI and SIM/Scan mode with time windows and internal calibration (one quantifier, up to two qualifiers).

### 4.13. Statistical Analysis

All statistics were performed with GraphPad Prism (version 9.2.0 for Windows, GraphPad Software, San Diego, CA, USA). A one-way or two-way analysis of variance (ANOVA) was conducted to examine differences in binding between groups, with a significance level set to α = 0.05. Post hoc tests were performed using Tukey’s honestly significant difference (HSD) test where appropriate. Statistical analysis for plasma and feces metabolites and correlations between feces, plasma metabolites, cytokines, microbiota, liver parameters were additionally performed using MetaboAnalyst 5.0 online platform [95]. Correlation testing between microbiota parameters, liver weight, fat content, and gene expression was performed using the Spearman test, correcting for multiple testing with the Benjamini–Hochberg test. Kruskal–Wallis test was used to identify differences between treatments for alpha-diversity and liver parameters using an R package.

## Figures and Tables

**Table 1:** Liver gene expression selected primer panel.

Primer Name	Target	Sequence
b-Act-F	beta-Actin	5′-ccc gcg agt aca acc ttc t-3′
b-Act-R	beta-Actin	5′-cgt cat cca tgg cga act-3′
HPRT-F	HPRT	5′-tag cac ctc ctc cgc cag-3′
HPRT-R	HPRT	5′-cac taa tca cga cgc tgg ga-3′
FAS-F	FAS	5′-ggc cac ctc agt cct gtt at-3′
FAS-R	FAS	5′-agg gtc cag cta gag ggt aca-3′
rFABP1-F	FABP1	5′-ctt ctc cgg caa gta cca ag-3′
rFABP1-R	FABP1	5′-ttc cct ttc tgg atg agg tc-3′
rLPL-1294-F	LPL	5′-aca gtg gct gag aac a-3′
rLPL-1445-R	LPL	5′-tct gac cag cta gga g-3′
PPARa-180-F	PPAR alpha	5′-CACAGCGTGGTGCATTTGG-3′
PPARa-412-R	PPAR alpha	5′-GAGAGAGGACAGATGGGGCT-3′
SREBP1-F	SREBP1	5′-gta cag cgt ggc tgg gaa c-3′
SREBP1-R	SREBP1	5′-ggc tga gcg ata cag ttc aa-3′

## Data Availability

Sequencing data (16S RNA gene, fecal microbiota) will be available from the end of March 2022 on the ENA website (accession number PRJEB49286).

## Supplementary Materials

The following are available online at https://www.mdpi.com/article/10.3390/ijms23031126/s1.

## References

[1] Yang Y.-J., Ni Y.-H. (2019). Gut microbiota and pediatric obesity/non-alcoholic fatty liver disease. J. Formos. Med. Assoc..

[2] Singer-Englar T., Barlow G., Mathur R. (2019). Obesity, diabetes, and the gut microbiome: An updated review. Expert Rev. Gastroenterol. Hepatol..

[3] Pradhan A.D., Manson J.E., Rifai N., Buring J.E., Ridker P.M. (2001). C-reactive protein, interleukin 6, and risk of developing type 2 diabetes mellitus. JAMA-J. Am. Med. Assoc..

[4] Jacka F.N., Cherbuin N., Anstey K.J., Sachdev P., Butterworth P. (2015). Western diet is associated with a smaller hippocampus: A longitudinal investigation. BMC Med..

[5] Andersen C.J., Murphy K.E., Fernandez M.L. (2016). Impact of Obesity and Metabolic Syndrome on Immunity. Adv. Nutr..

[6] Gaspar J.M., Baptista F.I., Paula Macedo M., Ambrosio A.F. (2016). Inside the Diabetic Brain: Role of Different Players Involved in Cognitive Decline. ACS Chem. Neurosci..

[7] Hotamisligil G.S. (2017). Inflammation, metaflammation and immunometabolic disorders. Nature.

[8] Pinhel M.A.S., Noronha N.Y., Nicoletti C.F., Pereira V.A.B., de Oliveira B.A.P., Cortes-Oliveira C., Salgado W., Barbosa F., Marchini J.S., Souza D.R.S. (2020). Changes in DNA Methylation and Gene Expression of Insulin and Obesity-Related GenePIK3R1after Roux-en-Y Gastric Bypass. Int. J. Mol. Sci..

[9] Hu F.B., van Dam R.M., Liu S. (2001). Diet and risk of Type II diabetes: The role of types of fat and carbohydrate. Diabetologia.

[10] Di Rienzi S.C., Britton R.A. (2020). Adaptation of the Gut Microbiota to Modern Dietary Sugars and Sweeteners. Adv. Nutr..

[11] Shalev D., Arbuckle M.R. (2017). Metabolism and Memory: Obesity, Diabetes, and Dementia. Biol. Psychiatry.

[12] Graham L.C., Harder J.M., Soto I., de Vries W.N., John S.W.M., Howell G.R. (2016). Chronic consumption of a western diet induces robust glial activation in aging mice and in a mouse model of Alzheimer’s disease. Sci. Rep..

[13] Almby K.E., Lundqvist M.H., Abrahamsson N., Kvernby S., Fahlstrom M., Pereira M.J., Gingnell M., Karlsson F.A., Fanni G., Sundbom M. (2021). Effects of Gastric Bypass Surgery on the Brain: Simultaneous Assessment of Glucose Uptake, Blood Flow, Neural Activity, and Cognitive Function During Normo- and Hypoglycemia. Diabetes.

[14] Khayyatzadeh S.S., Bagherniya M., Fazeli M., Khorasanchi Z., Bidokhti M.S., Ahmadinejad M., Khoshmohabbat S., Arabpour M., Afkhamizadeh M., Ferns G.A. (2018). A Western dietary pattern is associated with elevated level of high sensitive C-reactive protein among adolescent girls. Eur. J. Clin. Investig..

[15] Swinburn B.A., Sacks G., Hall K.D., McPherson K., Finegood D.T., Moodie M.L., Gortmaker S.L. (2011). Obesity 1 The global obesity pandemic: Shaped by global drivers and local environments. Lancet.

[16] World Health Organization Obesity and Overweight. https://www.who.int/news-room/fact-sheets/detail/obesity-and-overweight/.

[17] Apovian C.M., Aronne L.J., Bessesen D.H., McDonnell M.E., Murad M.H., Pagotto U., Ryan D.H., Still C.D. (2015). Pharmacological Management of Obesity: An Endocrine Society Clinical Practice Guideline. J. Clin. Endocrinol. Metab..

[18] Bauer K., Lau T., Schwille-Kiuntke J., Schild S., Hauner H., Stengel A., Zipfel S., Mack I. (2020). Conventional weight loss interventions across the different BMI obesity classes: A systematic review and quantitative comparative analysis. Eur. Eat. Disord. Rev..

[19] Abdeen G., le Roux C.W. (2016). Mechanism Underlying the Weight Loss and Complications of Roux-en-Y Gastric Bypass. Review. Obes. Surg..

[20] Lutz T.A., Bueter M. (2016). The Use of Rat and Mouse Models in Bariatric Surgery experiments. Front. Nutr..

[21] Olivo G., Zhou W., Sundbom M., Zhukovsky C., Hogenkamp P., Nikontovic L., Stark J., Wiemerslage L., Larsson E.M., Benedict C. (2017). Resting-state brain connectivity changes in obese women after Roux-en-Y gastric bypass surgery: A longitudinal study. Sci. Rep..

[22] Näslund E., Melin I., Grybäck P., Hägg A., Hellström P.M., Jacobsson H., Theodorsson E., Rössner S., Backman L. (1997). Reduced food intake after jejunoileal bypass: A possible association with prolonged gastric emptying and altered gut hormone patterns. Am. J. Clin. Nutr..

[23] Olbers T., Björkman S., Lindroos A., Maleckas A., Lönn L., Sjöström L., Lönroth H. (2006). Body composition, dietary intake, and energy expenditure after laparoscopic Roux-en-Y gastric bypass and laparoscopic vertical banded gastroplasty: A randomized clinical trial. Ann. Surg..

[24] Zheng H., Shin A.C., Lenard N.R., Townsend R.L., Patterson L.M., Sigalet D.L., Berthoud H.R. (2009). Meal patterns, satiety, and food choice in a rat model of Roux-en-Y gastric bypass surgery. Am. J. Physiol. Regul. Integr. Comp. Physiol..

[25] Schauer P.R., Mingrone G., Ikramuddin S., Wolfe B. (2016). Clinical Outcomes of Metabolic Surgery: Efficacy of Glycemic Control, Weight Loss, and Remission of Diabetes. Diabetes Care.

[26] Schauer P.R., Bhatt D.L., Kirwan J.P., Wolski K., Aminian A., Brethauer S.A., Navaneethan S.D., Singh R.P., Pothier C.E., Nissen S.E. (2017). Bariatric Surgery versus Intensive Medical Therapy for Diabetes—5-Year Outcomes. N. Engl. J. Med..

[27] Kwon I.G., Kang C.W., Park J.P., Oh J.H., Wang E.K., Kim T.Y., Sung J.S., Park N., Lee Y.J., Sung H.J. (2021). Serum glucose excretion after Roux-en-Y gastric bypass: A potential target for diabetes treatment. Gut.

[28] Saeidi N., Meoli L., Nestoridi E., Gupta N.K., Kvas S., Kucharczyk J., Bonab A.A., Fischman A.J., Yarmush M.L., Stylopoulos N. (2013). Reprogramming of Intestinal Glucose Metabolism and Glycemic Control in Rats After Gastric Bypass. Science.

[29] Cavin J.B., Couvelard A., Lebtahi R., Ducroc R., Arapis K., Voitellier E., Cluzeaud F., Gillard L., Hourseau M., Mikail N. (2016). Differences in Alimentary Glucose Absorption and Intestinal Disposal of Blood Glucose After Roux-en-Y Gastric Bypass vs. Sleeve Gastrectomy. Gastroenterology.

[30] Grayson B.E., Schneider K.M., Woods S.C., Seeley R.J. (2013). Improved Rodent Maternal Metabolism but Reduced Intrauterine Growth after Vertical Sleeve Gastrectomy. Sci. Transl. Med..

[31] Fernandes-Lima F., Monte T., Nascimento F.A.D., Gregorio B.M. (2015). Short Exposure to a High-Sucrose Diet and the First ‘Hit’ of Nonalcoholic Fatty Liver Disease in Mice. Cells Tissues Organs.

[32] Palleja A., Kashani A., Allin K.H., Nielsen T., Zhang C., Li Y., Brach T., Liang S., Feng Q., Jorgensen N.B. (2016). Roux-en-Y gastric bypass surgery of morbidly obese patients induces swift and persistent changes of the individual gut microbiota. Genome Med..

[33] Liou A.P., Paziuk M., Luevano J.-M., Machineni S., Turnbaugh P.J., Kaplan L.M. (2013). Conserved Shifts in the Gut Microbiota Due to Gastric Bypass Reduce Host Weight and Adiposity. Sci. Transl. Med..

[34] Kirchner H., Nylen C., Laber S., Barres R., Yan J., Krook A., Zierath J.R., Naslund E. (2014). Altered promoter methylation of PDK4, IL1 B, IL6, and TNF after Roux-en Y gastric bypass. Surg. Obes. Relat. Dis..

[35] Gupta A., Osadchiy V., Mayer E.A. (2020). Brain-gut-microbiome interactions in obesity and food addiction. Nat. Rev. Gastroenterol. Hepatol..

[36] Luo P., Yu H., Zhao X., Bao Y., Hong C.S., Zhang P., Tu Y., Yin P., Gao P., Wei L. (2016). Metabolomics Study of Roux-en-Y Gastric Bypass Surgery (RYGB) to Treat Type 2 Diabetes Patients Based on Ultraperformance Liquid Chromatography-Mass Spectrometry. J. Proteome Res..

[37] Cerreto M., Santopaolo F., Gasbarrini A., Pompili M., Ponziani F.R. (2021). Bariatric Surgery and Liver Disease: General Considerations and Role of the Gut-Liver Axis. Nutrients.

[38] Seyfried F., Phetcharaburanin J., Glymenaki M., Nordbeck A., Hankir M., Nicholson J.K., Holmes E., Marchesi J.R., Li J.V. (2021). Roux-en-Y gastric bypass surgery in Zucker rats induces bacterial and systemic metabolic changes independent of caloric restriction-induced weight loss. Gut Microbes.

[39] Ha J., Kwon Y., Park S. (2021). Metabolomics in Bariatric Surgery: Towards Identification of Mechanisms and Biomarkers of Metabolic Outcomes. Obes. Surg..

[40] Keller A., Della Torre S.B. (2015). Sugar-Sweetened Beverages and Obesity among Children and Adolescents: A Review of Systematic Literature Reviews. Child. Obes..

[41] Ritze Y., Bárdos G., D’Haese J.G., Ernst B., Thurnheer M., Schultes B., Bischoff S.C. (2014). Effect of High Sugar Intake on Glucose Transporter and Weight Regulating Hormones in Mice and Humans. PLoS ONE.

[42] Hao Z., Townsend R.L., Mumphrey M.B., Morrison C.D., Munzberg H., Berthoud H.-R. (2017). RYGB Produces more Sustained Body Weight Loss and Improvement of Glycemic Control Compared with VSG in the Diet-Induced Obese Mouse Model. Obes. Surg..

[43] Baheeg M., El-Din M.T., Labib M.F., Elgohary S.A., Hasan A. (2021). Long-term durability of weight loss after bariatric surgery; a retrospective study. Int. J. Surg. Open.

[44] Guo Y., Huang Z.-P., Liu C.-Q., Qi L., Sheng Y., Zou D.-J. (2018). Modulation of the gut microbiome: A systematic review of the effect of bariatric surgery. Eur. J. Endocrinol..

[45] Cook J., Lehne C., Weiland A., Archid R., Ritze Y., Bauer K., Zipfel S., Penders J., Enck P., Mack I. (2020). Gut Microbiota, Probiotics and Psychological States and Behaviors after Bariatric Surgery-A Systematic Review of Their Interrelation. Nutrients.

[46] Strandwitz P., Kim K.H., Terekhova D., Liu J.K., Sharma A., Levering J., McDonald D., Dietrich D., Ramadhar T.R., Lekbua A. (2019). GABA-modulating bacteria of the human gut microbiota. Nat. Microbiol..

[47] Strandwitz P. (2018). Neurotransmitter modulation by the gut microbiota. Brain Res..

[48] Chen O., Mah E., Dioum E., Marwaha A., Shanmugam S., Malleshi N., Sudha V., Gayathri R., Unnikrishnan R., Anjana R.M. (2021). The Role of Oat Nutrients in the Immune System: A Narrative Review. Nutrients.

[49] Krantis A. (2000). GABA in the mammalian enteric nervous system. News Physiol. Sci..

[50] Inotsuka R., Uchimura K., Yamatsu A., Kim M., Katakura Y. (2020). Gamma-Aminobutyric acid (GABA) activates neuronal cells by inducing the secretion of exosomes from intestinal cells. Food Funct..

[51] Auteri M., Zizzo M.G., Serio R. (2015). The GABAergic System and the Gastrointestinal Physiopathology. Curr. Pharm. Des..

[52] Nicholson J.K., Holmes E., Kinross J., Burcelin R., Gibson G., Jia W., Pettersson S. (2012). Host-Gut Microbiota Metabolic Interactions. Science.

[53] Lagkouvardos I., Lesker T.R., Hitch T.C.A., Galvez E.J.C., Smit N., Neuhaus K., Wang J., Baines J.F., Abt B., Stecher B. (2019). Sequence and cultivation study of Muribaculaceae reveals novel species, host preference, and functional potential of this yet undescribed family. Microbiome.

[54] Rowland I., Gibson G., Heinken A., Scott K., Swann J., Thiele I., Tuohy K. (2018). Gut microbiota functions: Metabolism of nutrients and other food components. Eur. J. Nutr..

[55] Wang G., Wang Q., Bai J., Zhao N., Wang Y., Zhou R., Kong W., Zeng T., Tao K., Wang G. (2020). Upregulation of Intestinal NLRP6 Inflammasomes After Roux-en-Y Gastric Bypass Promotes Gut Immune Homeostasis. Obes. Surg..

[56] Zhu Y., Jameson E., Crosatti M., Schaefer H., Rajakumar K., Bugg T.D.H., Chen Y. (2014). Carnitine metabolism to trimethylamine by an unusual Rieske-type oxygenase from human microbiota. Proc. Natl. Acad. Sci. USA.

[57] Craciun S., Balskus E.P. (2012). Microbial conversion of choline to trimethylamine requires a glycyl radical enzyme. Proc. Natl. Acad. Sci. USA.

[58] Chen Y.-R., Fang S.-T., Liu H.-Y., Zheng H.-M., He Y., Chen Z.-W., Chen M.-X., Zhang G.-X., Zhou H.-W. (2018). Degradation of trimethylamine in vitro and in vivo by Enterococcus faecalis isolated from healthy human gut. Int. Biodeterior. Biodegrad..

[59] Liu Y., Dai M. (2020). Trimethylamine N-Oxide Generated by the Gut Microbiota Is Associated with Vascular Inflammation: New Insights into Atherosclerosis. Mediat. Inflamm..

[60] Yang S., Li X., Yang F., Zhao R., Pan X., Liang J., Tian L., Li X., Liu L., Xing Y. (2019). Gut Microbiota-Dependent Marker TMAO in Promoting Cardiovascular Disease: Inflammation Mechanism, Clinical Prognostic, and Potential as a Therapeutic Target. Front. Pharmacol..

[61] Rath S., Heidrich B., Pieper D.H., Vital M. (2017). Uncovering the trimethylamine-producing bacteria of the human gut microbiota. Microbiome.

[62] Wu Q., Li J.V., Seyfried F., le Roux C.W., Ashrafian H., Athanasiou T., Fenske W., Darzi A., Nicholson J.K., Holmes E. (2015). Metabolic phenotype-microRNA data fusion analysis of the systemic consequences of Roux-en-Y gastric bypass surgery. Int. J. Obes..

[63] Siddik M.A.B., Shin A.C. (2019). Recent Progress on Branched-Chain Amino Acids in Obesity, Diabetes, and Beyond. Endocrinol. Metab..

[64] White P.J., Lapworth A.L., An J., Wang L., McGarrah R.W., Stevens R.D., Ilkayeva O., George T., Muehlbauer M.J., Bain J.R. (2016). Branched-chain amino acid restriction in Zucker-fatty rats improves muscle insulin sensitivity by enhancing efficiency of fatty acid oxidation and acyl-glycine export. Mol. Metab..

[65] Rosenzweig A., Blenis J., Gomes A.P. (2018). Beyond the Warburg Effect: How Do Cancer Cells Regulate One-Carbon Metabolism?. Front. Cell Dev. Biol..

[66] Alves A., Bassot A., Bulteau A.-L., Pirola L., Morio B. (2019). Glycine Metabolism and Its Alterations in Obesity and Metabolic Diseases. Nutrients.

[67] Lim P.S., Chang Y.-K., Wu T.-K. (2019). Serum Lipopolysaccharide-Binding Protein is Associated with Chronic Inflammation and Metabolic Syndrome in Hemodialysis Patients. Blood Purif..

[68] Parlesak A., Schaeckeler S., Moser L., Bode C. (2007). Conjugated primary bile salts reduce permeability of endotoxin through intestinal epithelial cells and synergize with phosphatidylcholine in suppression of inflammatory cytokine production. Crit. Care Med..

[69] Lin T.-L., Shu C.-C., Chen Y.-M., Lu J.-J., Wu T.-S., Lai W.-F., Tzeng C.-M., Lai H.-C., Lu C.-C. (2020). Like Cures Like: Pharmacological Activity of Anti-Inflammatory Lipopolysaccharides From Gut Microbiome. Front. Pharmacol..

[70] Whipp A.M., Vuoksimaa E., Korhonen T., Pool R., But A., Ligthart L., Hagenbeek F.A., Bartels M., Bogl L.H., Pulkkinen L. (2021). Ketone body 3-hydroxybutyrate as a biomarker of aggression. Sci. Rep..

[71] Ghoshal S., Witta J., Zhong J., de Villiers W., Eckhardt E. (2009). Chylomicrons promote intestinal absorption of lipopolysaccharides. J. Lipid Res..

[72] Halinski L.P., Pakiet A., Jablonska P., Kaska L., Proczko-Stepaniak M., Slominska E., Sledzinski T., Mika A. (2020). One Anastomosis Gastric Bypass Reconstitutes the Appropriate Profile of Serum Amino Acids in Patients with Morbid Obesity. J. Clin. Med..

[73] Kindt A., Liebisch G., Clavel T., Haller D., Hoermannsperger G., Yoon H., Kolmeder D., Sigruener A., Krautbauer S., Seeliger C. (2018). The gut microbiota promotes hepatic fatty acid desaturation and elongation in mice. Nat. Commun..

[74] Weitkunat K., Schumann S., Nickel D., Hornemann S., Petzke K.J., Schulze M.B., Pfeiffer A.F.H., Klaus S. (2017). Odd-chain fatty acids as a biomarker for dietary fiber intake: A novel pathway for endogenous production from propionate. Am. J. Clin. Nutr..

[75] Yang X., Sun G.Y., Eckert G.P., Lee J.C.M. (2014). Cellular Membrane Fluidity in Amyloid Precursor Protein Processing. Mol. Neurobiol..

[76] Vlaeminck B., Fievez V., Cabrita A.R.J., Fonseca A.J.M., Dewhurst R.J. (2006). Factors affecting odd- and branched-chain fatty acids in milk: A review. Anim. Feed. Sci. Technol..

[77] Fonteh A.N., Cipolla M., Chiang J., Arakaki X., Harrington M.G. (2014). Human Cerebrospinal Fluid Fatty Acid Levels Differ between Supernatant Fluid and Brain-Derived Nanoparticle Fractions, and Are Altered in Alzheimer’s Disease. PLoS ONE.

[78] Lam T.K.T., Pocai A., Gutierrez-Juarez R., Obici S., Bryan J., Aguilar-Bryan L., Schwartz G.J., Rossetti L. (2005). Hypothalamic sensing of circulating fatty acids is required for glucose homeostasis. Nat. Med..

[79] Gonzalez-Hernandez T., Barroso-Chinea P., de la Cruz M.A.P., Valera P., Dopico J.G., Rodriguez M. (2002). Response of gabaergic cells in the deep mesencephalic nucleus to dopaminergic cell degeneration: An electrophysiological and in situ hybridization study. Neuroscience.

[80] Hankir M.K., Seyfried F., Miras A.D., Cowley M.A. (2018). Brain Feeding Circuits after Roux-en-Y Gastric Bypass. Trends Endocrinol. Metab..

[81] Singh Y., Trautwein C., Dhariwal A., Salker M.S., Alauddin M., Zizmare L., Pelzl L., Feger M., Admard J., Casadei N. (2020). DJ-1 (Park7) affects the gut microbiome, metabolites and the development of innate lymphoid cells (ILCs). Sci. Rep..

[82] Bueter M., Abegg K., Seyfried F., Lutz T.A., le Roux C.W. (2012). Gastric Bypass Operation in Rats. J. Vis. Exp..

[83] Wehrl H.F., Hossain M., Lankes K., Liu C.-C., Bezrukov I., Martirosian P., Schickt F., Reischl G., Pichler B.J. (2013). Simultaneous PET-MRI reveals brain function in activated and resting state on metabolic, hemodynamic and multiple temporal scales. Nat. Med..

[84] Schiffer W.K., Mirrione M.M., Biegon A., Alexoff D.L., Patel V., Dewey S.L. (2006). Serial microPET measures of the metabolic reaction to a microdialysis probe implant. J. Neurosci. Methods.

[85] Ionescu T.M., Amend M., Hafiz R., Biswal B.B., Wehrl H.F., Herfert K., Pichler B.J. (2021). Elucidating the complementarity of resting-state networks derived from dynamic F-18 FDG and hemodynamic fluctuations using simultaneous small-animal PET/MRI. Neuroimage.

[86] Paxinos G., Watson C. (1998). The Rat Brain in Stereotaxic Coordinates.

[87] Caporaso J.G., Lauber C.L., Walters W.A., Berg-Lyons D., Lozupone C.A., Turnbaugh P.J., Fierer N., Knight R. (2011). Global patterns of 16S rRNA diversity at a depth of millions of sequences per sample. Proc. Natl. Acad. Sci. USA.

[88] Walters W., Hyde E.R., Berg-Lyons D., Ackermann G., Humphrey G., Parada A., Gilbert J.A., Jansson J.K., Caporaso J.G., Fuhrman J.A. (2016). Improved Bacterial 16S rRNA Gene (V4 and V4-5) and Fungal Internal Transcribed Spacer Marker Gene Primers for Microbial Community Surveys. Msystems.

[89] Kozich J.J., Westcott S.L., Baxter N.T., Highlander S.K., Schloss P.D. (2013). Development of a Dual-Index Sequencing Strategy and Curation Pipeline for Analyzing Amplicon Sequence Data on the MiSeq Illumina Sequencing Platform. Appl. Environ. Microbiol..

[90] Callahan B.J., McMurdie P.J., Holmes S.P. (2017). Exact sequence variants should replace operational taxonomic units in marker-gene data analysis. ISME J..

[91] McMurdie P.J., Holmes S. (2013). phyloseq: An R Package for Reproducible Interactive Analysis and Graphics of Microbiome Census Data. PLoS ONE.

[92] Segata N., Izard J., Waldron L., Gevers D., Miropolsky L., Garrett W.S., Huttenhower C. (2011). Metagenomic biomarker discovery and explanation. Genome Biol..

[93] Ecker J., Scherer M., Schmitz G., Liebisch G. (2012). A rapid GC-MS method for quantification of positional and geometric isomers of fatty acid methyl esters. Journal of chromatography. B. Anal. Technol. Biomed. Life Sci..

[94] Gille A., Hollenbach R., Trautmann A., Gomez M.R., Kruger R., Bischoff S.C., Posten C., Briviba K. (2020). Lipophilic compounds, but not fucoxanthin, mediate the genotoxic effect of photoautotrophic grown Phaeodactylum tricomutum in Caco-2 and HT-29 cells. J. Funct. Foods.

[95] Chong J., Wishart D.S., Xia J. (2019). Using MetaboAnalyst 4.0 for Comprehensive and Integrative Metabolomics Data Analysis. Curr. Protoc. Bioinform..

