# Supplementary material for: Roux-En-Y Gastric Bypass (RYGB) Surgery during High Liquid Sucrose Diet Leads to Gut Microbiota-Related Systematic Alterations
Source: Int J Mol Sci. 2022 Jan 20;23(3):1126. doi: 10.3390/ijms23031126 (PMC8835548; doi:10.3390/ijms23031126)
Supplement: Supplementary file 1 [file ijms-23-01126-s001.zip › ijms-1546908 supplementary corrected.pdf]

# Supplementary Material

1. Supplementary figures S1 – S8
2. Supplementary tables ST1 – ST3

# Roux-en-Y Gastric Bypass (RYGB) Surgery During High Liquid Sucrose Diet Leads to Gut Microbiota-Related Systematic Alterations

Laimdota Zizmare[1], Christina N. Boyle[2], Sabrina Buss[1], Sandrine Louis[3], Laura Kuebler[1], Ketki Mulay[4], Ralf Krüger[3], Lara Steinhauer[3], Isabelle Mack[5], Manuel Rodriguez Gomez[3], Kristina Herfert[1], Yvonne Ritze[4,*], Christoph Trautwein[1]

[1]Werner Siemens Imaging Center, Department of Preclinical Imaging and Radiopharmacy, Eberhard Karls University of Tübingen, University Hospital Tübingen, Röntgenweg 13, 72076, Tübingen, Germany;

[2] Institute of Veterinary Physiology, University of Zurich, Winterthurerstraße 260, CH-8057, Zurich, Switzerland;

[3] Department of Physiology and Biochemistry of Nutrition, Max Rubner-Institut, Haid-und-Neu-Straße 9, 76131, Karlsruhe, Germany;

[4] Department of Medical Psychology and Behavioral Neurobiology, Eberhard Karls University of Tübingen, Silcherstraße 5, 72076, Tübingen, Germany;

[5] Department of Psychosomatic Medicine and Psychotherapy, University Hospital Tübingen, Osianderstraße 5, 72076, Tübingen, Germany.

[*]Corresponding author: Yvonne Ritze, contact@yvonne-ritze.com

# Supplementary Figure S1



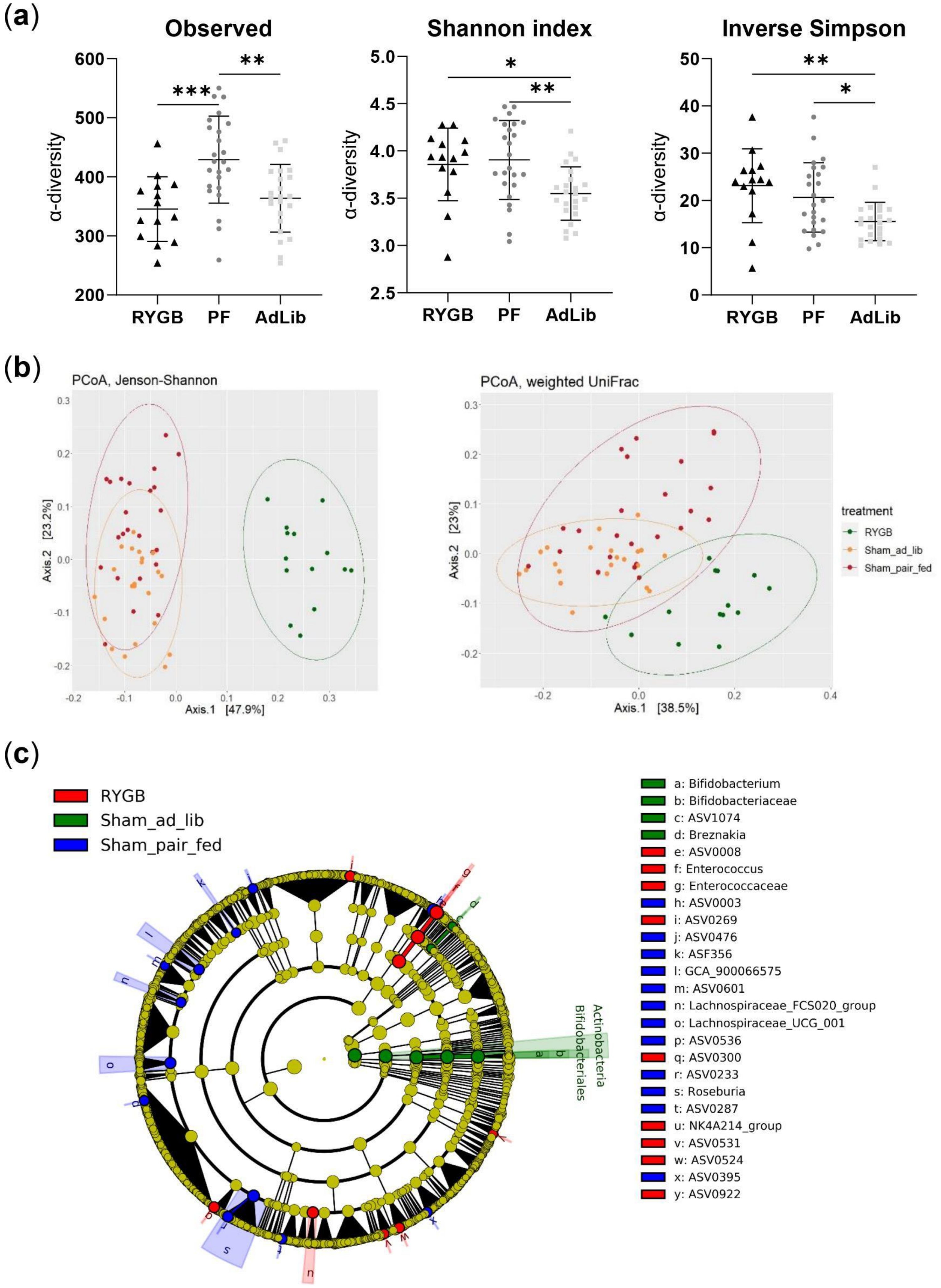


**Supplementary Figure S1: Microbiota analysis.** (**a**) α-diversity, the microbial community in AdLib animals was less diverse than in the other groups (diversity as the observed number of species, Shannon index and inverse Simpson). (**b**) β-diversity, principal coordinate analysis (PCoA) illustrating the separation of RYGB samples from the 2 other groups and showing the changes in the microbiota composition after surgery (PCoA based on either the Jenson-Shannon distance or the weighted UniFrac distance; each dot represents the whole gut microbiota from each animal at feces collection time). (**c**) LefSe analysis indicates the significant marker of each group represented in a phylogenetic cladogram. Pooled data was used from feces collection at four and eight weeks post-surgery, and at the day of euthanasia; *p*-values: *** < 0.001, ** < 0.01, * < 0.05.

**Supplementary Figure S2**

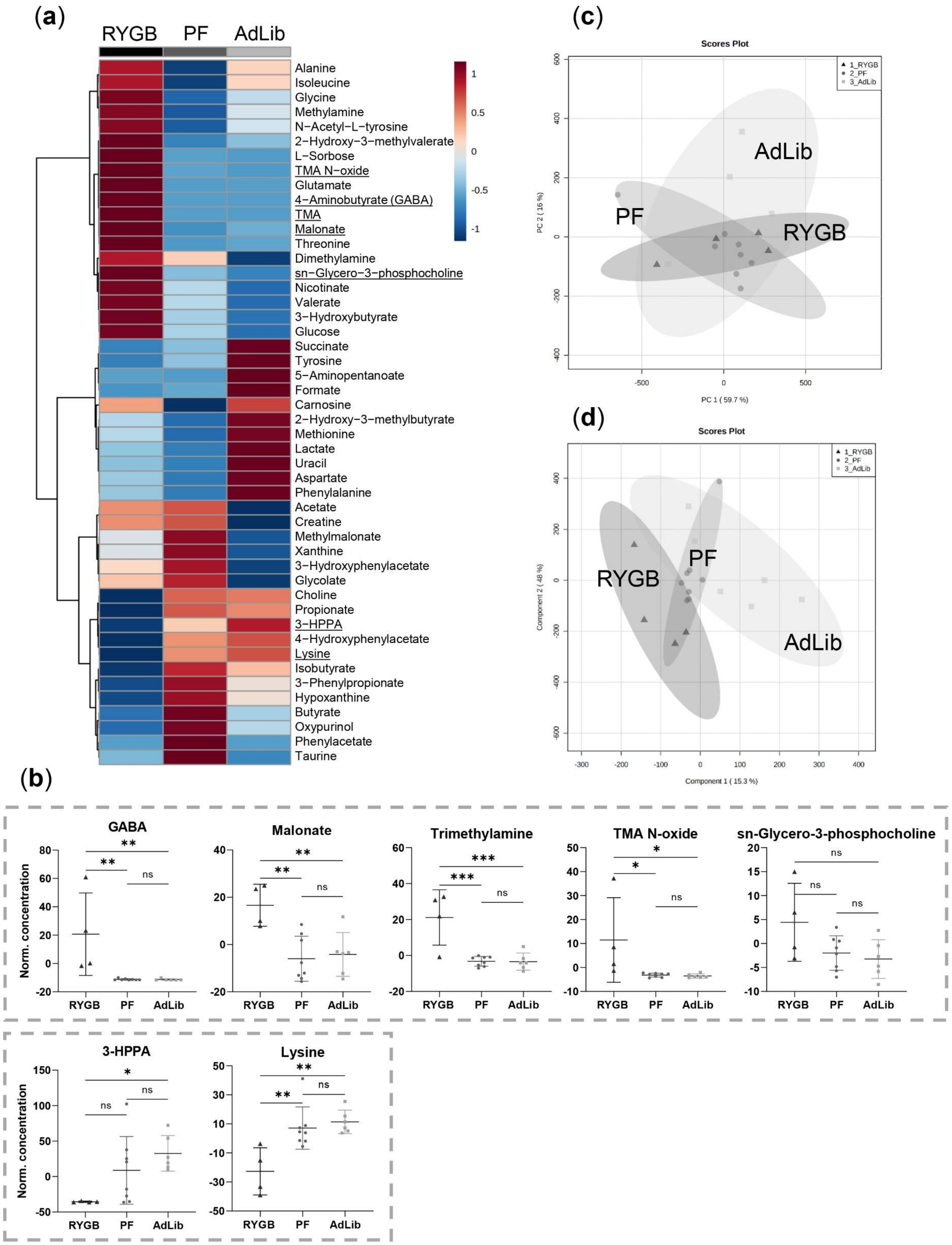

**Supplementary Figure S2: Feces metabolomics four weeks after RYGB/sham surgery.** (**a**) Averaged heat map illustrating all the quantified feces metabolites with their relative concentration increase (red) or decrease (blue). (**b**) Individual metabolite box plots. Decreased 3-hydroxyphenylpropionate (3-HPPA) and lysine in RYGB. Increased 4-aminobutyrate (GABA), malonate, trimethylamine (TMA) and TMA N-oxide in RYGB. Statistical significance based on one-way ANOVA with illustrated individual animal data points. (**c**) Principal component analysis (PCA) of feces samples 4 weeks after surgery. (**d**) Partial least squares discriminant analysis (PLSDA) regression model illustrating the further group cluster separation. 95% confidence regions are illustrated as clouds around the individual data points. RYGB (n = 4) (one animal excluded due to technical limitation of poor spectra resolution) black triangles, PF (n = 8) dark grey circles, AdLib (n = 6) light grey squares, *p*-values: *** < 0.001, ** < 0.01, * < 0.05.

# Supplementary Figure S3

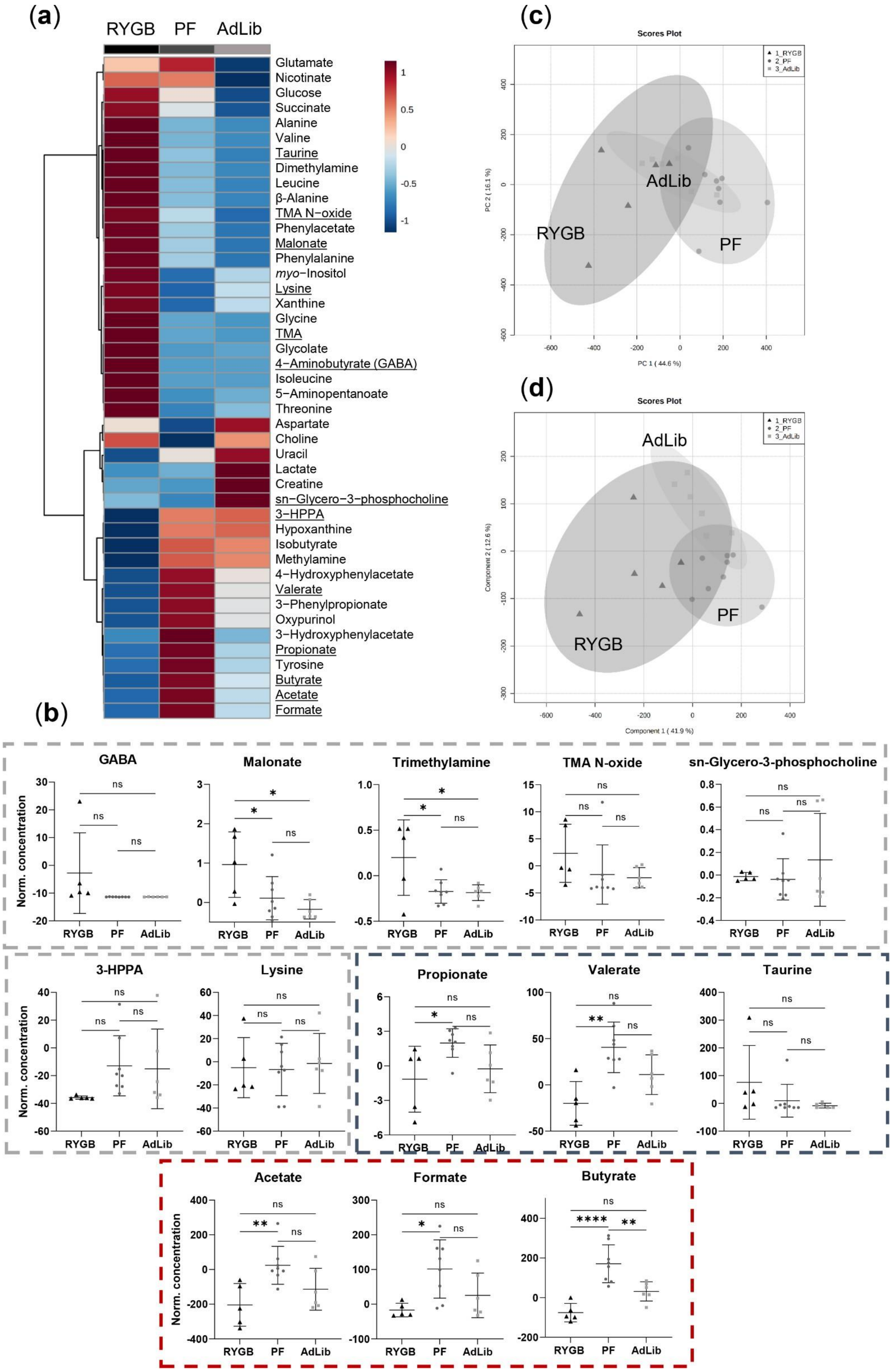

**Supplementary Figure S3: Metabolite analysis of feces collected on the day of euthanasia from the colon after RYGB/sham surgery.** (**a**) Averaged heat map illustrating all the quantified feces metabolites with their relative concentration increase (red) or decrease (blue). (**b**) Individual metabolite box plots. Grey box – feces metabolite patterns since week 4. Blue box – feces metabolite changes introduced at week 8. Red box – novel metabolite changes in feces collected on the day of euthanasia: decreased acetate, formate and butyrate in RYGB. Statistical significance based on one-way ANOVA with illustrated individual animal metabolite concentration as points. (**c**) Principal component analysis (PCA) of feces samples 4 weeks after surgery. (**d**) Partial least squares discriminant analysis (PLSDA) regression model illustrating the further group cluster separation with the RYGB sample sparsity. 95% confidence regions are illustrated as clouds around the individual data points. RYGB (n = 5) black triangles, PF (n = 8) dark grey dots, AdLib (n = 6) light grey squares, *p*-values: **** < 0.0001, ** < 0.01, * < 0.05.

# Supplementary Figure S7

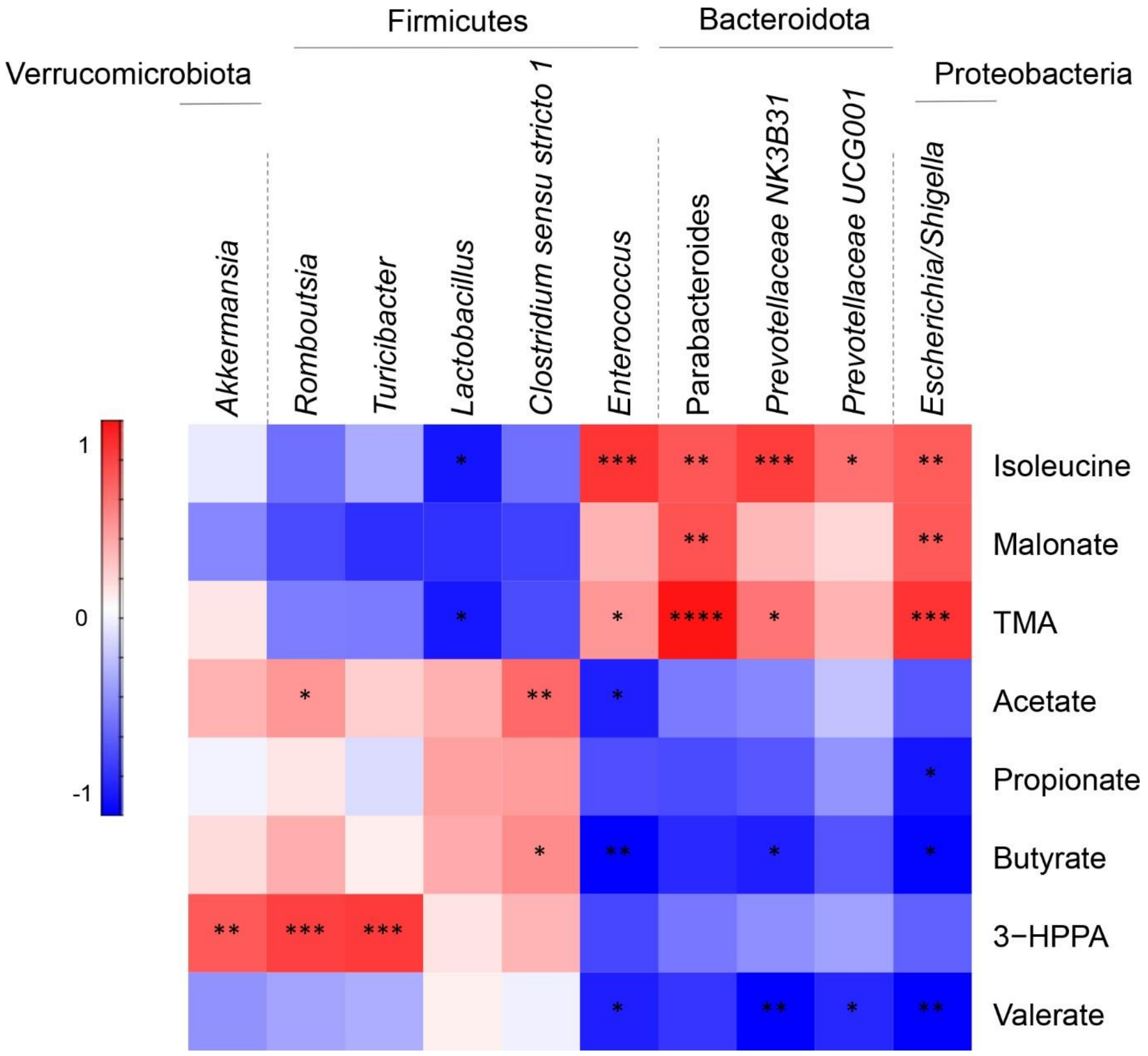


**Supplementary Figure S4: Feces microbiota and metabolomics correlation analysis.** Feces were collected directly from the colon on the day of euthanasia. 3-HPPA positively correlated with the relative abundance of *Turicibacter* and *Rombutsia*. TMA positively correlated with the relative abundance of *Escherichia/Shigella* and *Parabacteroides*. Isoleucine positively correlated with the relative abundance of *Prevotellaceae NK3B31* and *Enterococcus*. Color scheme illustrates the correlation values scaled between -1 and 1. Red color indicates a positive correlation, blue – negative correlation. Statistical significance of these correlations illustrated as *p*-values: **** < 0.0001, *** < 0.001, ** < 0.01, * < 0.05. Correlation analysis involves all three group (RYGB, PF and AdLib) feature values using Pearson r distance measure for the correlation coefficient determination.

# Supplementary Figure S8



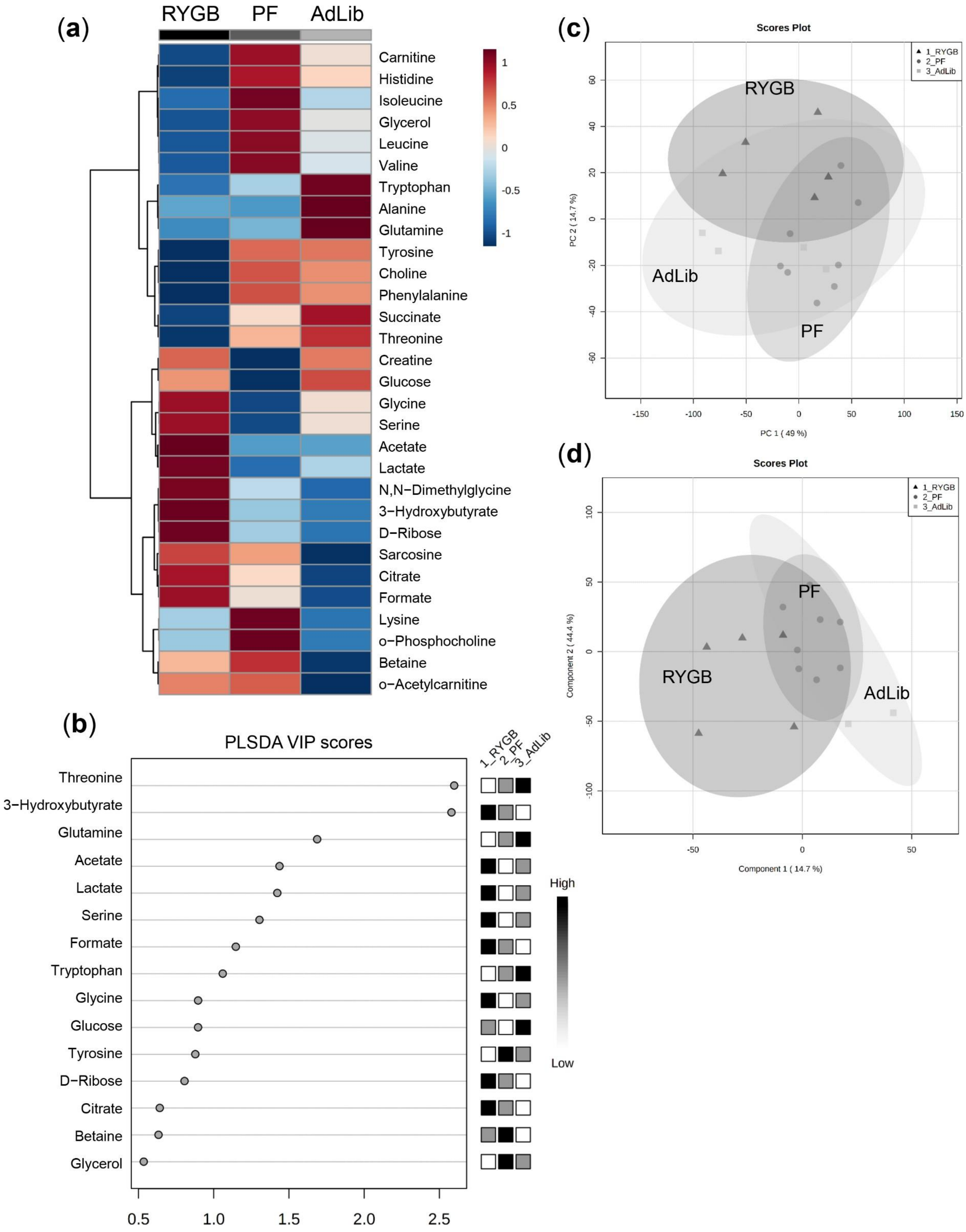


**Supplementary Figure S5: Plasma metabolomics.** (**a**) Averaged group metabolite concentration heat map illustrating all the quantified plasma metabolites with their relative concentration increase (red) or decrease (blue). (**b**) Partial least squares discriminant analysis (PLSDA) variable importance in projection (VIP) scores visualisation of most changing metabolites between the groups. (**c**) Principal component analysis (PCA) of plasma samples. (**d**) PLSDA regression model illustrating the further group cluster separation. 95% confidence regions are illustrated as clouds around the individual data points. RYGB (n = 5), PF (n = 8), AdLib (n = 6).

(a)

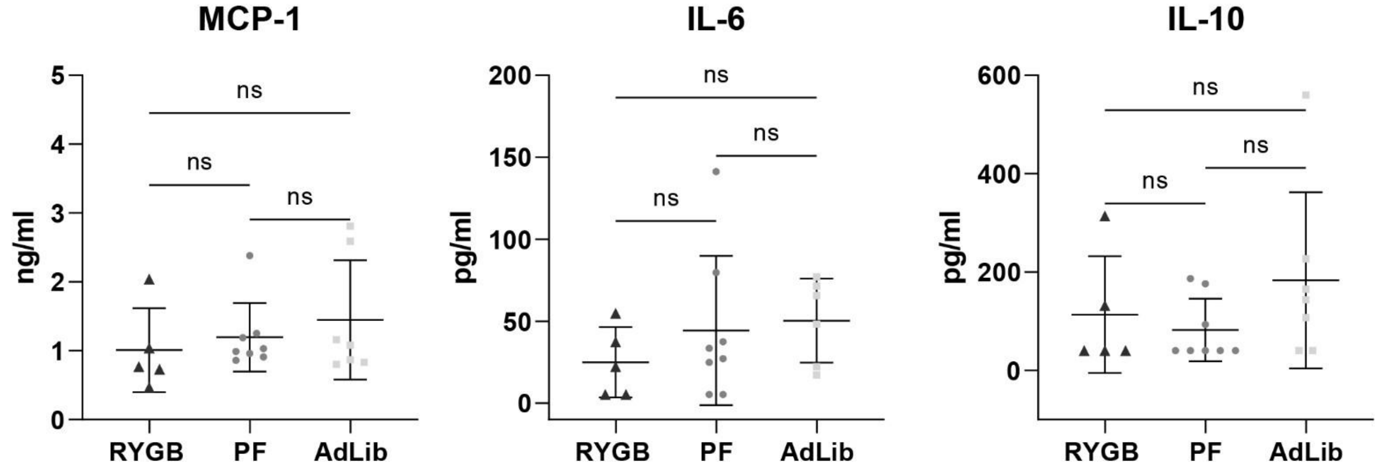


(b)

| | RYGB | PF | AdLib |
|---|---|---|---|
| CXCL2 (pg/ml) | below LOD | below LOD | below LOD |
| IL-1α (pg/ml) | below LOD | below LOD | below LOD |
| IFN-γ (pg/ml) | below LOD | below LOD | below LOD |
| TNF-α (pg/ml) | below LOD | below LOD | below LOD |

**Supplementary Figure S6: Cytokine profiling of rat plasma**. (**a**) Quantification of Monocyte Chemoattractant Protein-1 (MCP-1), Interleukin 6 (IL-6) and Interleukin 10 (IL-10), did not pass ordinary one-way ANOVA statistical significance threshold; (**b**) Chemokine ligand 2 (CXCL2), interleukin 1α (IL-1α), Interferon gamma (IFN-γ) and Tumour Necrosis Factor (TNF) were below the limit of detection (LOD). RYGB (n = 5) black triangles, PF (n = 8) dark grey dots, AdLib (n = 7) light grey squares.

**Supplementary figure S7**

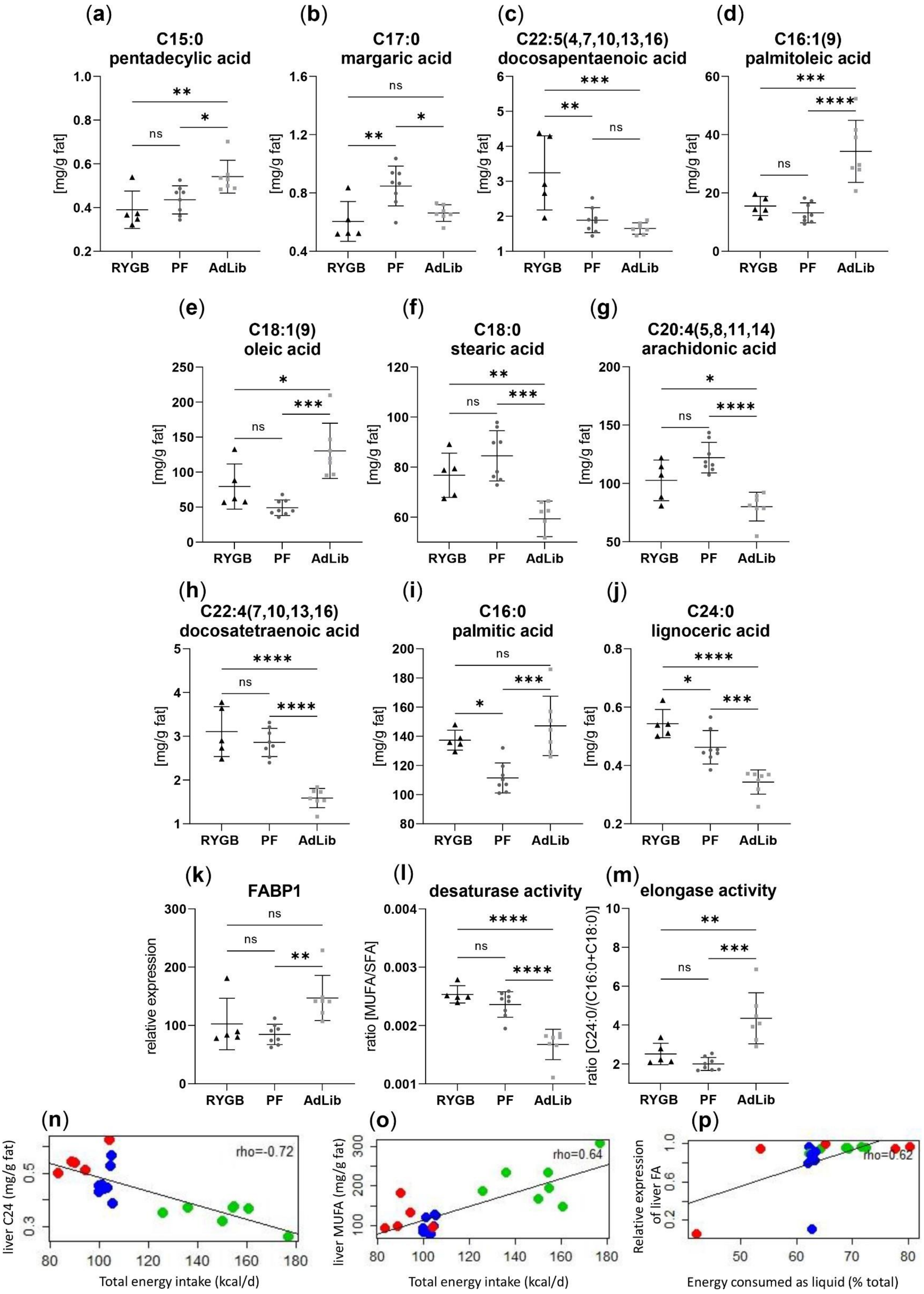

**Supplementary Figure S7: Hepatic lipid analysis.** Mass concentrations (mg/g fat) of hepatic lipid species upregulated in AdLib C14:0 (**a**), C15:0 (**b**), C16:1(9) (**c**), C18:1(9) (**d**); downregulated in AdLib C18:0 (**e**); C20:4(5,8,11,14) (**f**), C22:4(7,10,13,16) (**g**); downregulated in RYGB C18:1(11) (**h**); upregulated in RYGB C22:5(4,7,10,13,16) (**i**), C24:1(15) (**j**); downregulated in AdLib and slightly downregulated in PF C24:0 (**k**); upregulated in PF C17:0 (**l**); downregulated in RYGB C18:1(11) (**m**) compared to the other two groups. One-way ANOVA statistical significance test, RYGB (n = 5) black triangles, PF (n = 8) dark grey dots, AdLib (n = 7) light grey squares, *p*-values: **** < 0.0001, *** < 0.001, ** < 0.01, * < 0.05. (**n**) Correlations between the total energy intake (kcal/d) versus liver C24:0 (mg/g fat) (*p*=5.10-4; rho = -0.72) and (**o**) liver MUFA (mg/g fat) (*p*=0.0029; rho = 0.64); (**p**) correlation between the energy consumed as liquid (% total) versus relative expression of liver FAS (*p*= 0.0038; rho = 0.62). RYGB - red, PF - blue, AdLib - green.

## Supplementary figure S8

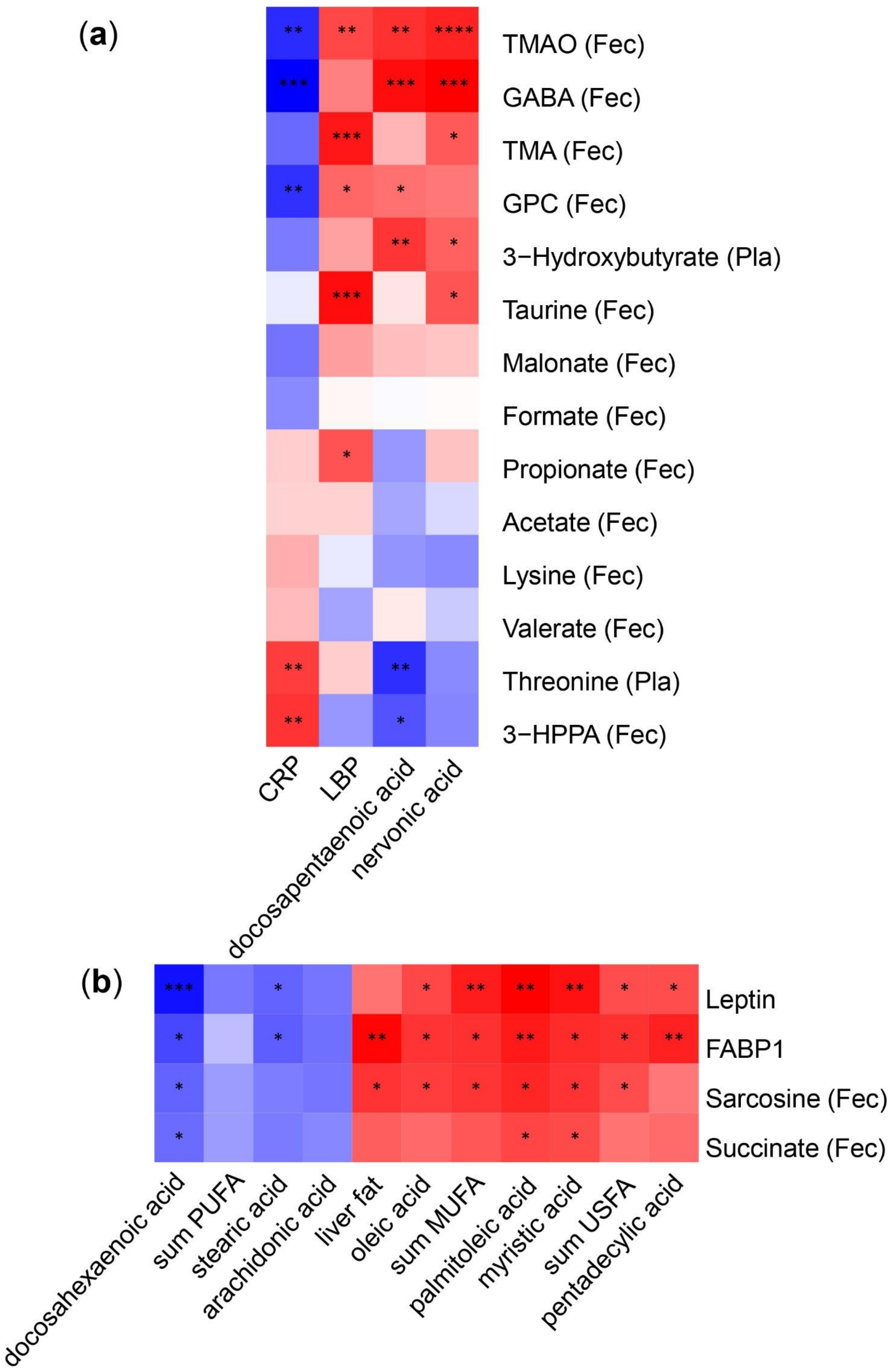


**Supplementary Figure S8: Correlation maps of metabolites, immune, hormonal and liver parameters**. (**a**) Sham vs. RYGB comparison significantly differing parameters, C22:5(4,7,10,13,16) (docosapentaenoic acid (osbond acid), PUFA, omega-6), C24:1(15) (nervonic acid, MUFA), (**b**) parameters similar between PF and RYGB (reduced food intake) compared to AdLib, C22:4(7,10,13,16) (docosatetraenoic acid, PUFA, omega-6), C16:1(9) (palmitoleic acid, MUFA), C14:0 (myristic acid, SFA), C15:0 (pentadecylic acid, SFA), C16:1(9) (palmitoleic acid, MUFA), *p*-values: **** < 0.0001, *** < 0.001, ** < 0.01, * < 0.05.

## Supplementary Tables

| Bregma -1.80 | | RYGB | PF | AdLib | *p*-value |
|---|---|---|---|---|---|
| **PVH** | Middle | 3.00±1.66 | 8.43± 4.55 | 5.50±2.88 | 0.44 |
| **CeC** | Right | 7.33±2.63 | 2.80±1.01 | 3.40±1.68 | 0.16 |
| | Left | 10.87±4.60 | 4.27±1.10 | 5.47±3.81 | 0.32 |
| **DeN** | Right | 2.07±1.18 | 0.93±0.43 | 2.53±1.28 | 0.48 |
| | Left | 1.20±0.45 | 0.83±0.40 | 6.07±2.34 | 0.02* |
| **BLA** | Right | 1.27±1.32 | 1.67±1.26 | 3.60 ± 1.82 | 0.44 |
| | Left | 0.87±0.60 | 4.40±3.68 | 2.87 ±2.45 | 0.57 |
| **layer 2 of cortex** | Right | 2.00±1.97 | 14.87±11.63 | 7.30±3.74 | 0.39 |
| | Left | 2.33±2.25 | 11.93±9.01 | 9.73±6.02 | 0.48 |

**Supplementary Table ST1: Average number of c-Fos labelled cells in different brain areas after eight weeks of high sucrose diet in rats** Average numbers were analyzed by counting cells on 3 distinct slices (60 µm thick) for every region. Data were analyzed by ANOVA test followed by a Tukey's post hoc test ($p$ <0.05). Data are means ± SEM (n = 5). PVH – paraventricular hypothalamic nucleus; CeC – central amygdala; DeN – dorsal endopiriform nucleus; BLA – basolateral amygdaloid nucleus, anterior.

| Bregma -1.80 | | RYGB | PF | AdLib | *p*-value |
|---|---|---|---|---|---|
| **PVH** | Middle | 33.73±13.61 | 55.27± 22.14 | 35.07±10.10 | 0.51 |
| **CeC** | Right | 41.20±9.36 | 33.53±13.12 | 41.87±14.44 | 0.84 |
| | Left | 39.60±7.69 | 51.20±14.34 | 43.53±21.57 | 0.84 |
| **DeN** | Right | 123.40±23.86 | 124.53±24.11 | 91.93±19.55 | 0.26 |
| | Left | 122.87±25.44 | 159.30±7.05 | 132.67±32.02 | 0.48 |
| **BLA** | Right | 161.80±21.55 | 172.33±52.96 | 148.47±37.41 | 0.89 |
| | Left | 184.27±14.95 | 179.93±41.91 | 182.80±32.78 | 0.99 |
| **layer 2 of cortex** | Right | 338.67±27.05 | 314.77±38.82 | 339.20±37.31 | 0.82 |
| | Left | 318.53±17.72 | 372.63±30.71 | 263.13±59.22 | 0.14 |

**Supplementary Table ST2: Average number of NeuN labeled cells in different brain areas after eight weeks of high sucrose diet in rats.** Average numbers were analyzed by counting cells on 3 distinct slices (60 µm thick) for every region. Data were analyzed by ANOVA test followed by a Tukey's post hoc test if significant (*P<0.05; n.s., non-significant). Data are means ± SEM (n = 5). PVH – paraventricular hypothalamic nucleus; CeC – central amygdala; DeN – dorsal endopiriform nucleus; BLA – basolateral amygdaloid nucleus, anterior.

| Bregma -1.80 | | Pair-fed | Ad-libitum | RYGB | p-value |
|---|---|---|---|---|---|
| **PVH** | Middle | 4.47±2.74 | 2.93±1.78 | 2.10±1.25 | 0.65 |
| **CeC** | Right | 0.87±0.62 | 2.13±1.07 | 2.93±0.88 | 0.21 |
| | Left | 1.67±0.54 | 2.80±1.92 | 4.73±2.48 | 0.43 |
| **DeN** | Right | 0.93±0.43 | 1.13±0.83 | 0.73±0.38 | 0.86 |
| | Left | 0.60±0.36 | 3.87±2.05 | 0.40±0.30 | 0.08 |
| **BLA** | Right | 1.60 ±1.24 | 2.80±1.97 | 0.08±0.08 | 0.31 |
| | Left | 3.87±3.12 | 2.73±2.31 | 0.40±0.45 | 0.49 |
| **layer 2 of cortex** | Right | 14.60±11.57 | 4.33±3.48 | 2.00±1.97 | 0.36 |
| | Left | 11.60±8.73 | 7.13±6.38 | 2.27±2.27 | 0.53 |

**Supplementary Table ST3**: **Average number of co-localized cells (labeled by both c-Fos and NeuN) in different brain areas after eight weeks of high sucrose diet in rats.** Average numbers were analyzed by counting cells on 3 distinct slices (60 µm thick) for every region. Data were analyzed by ANOVA test followed by a Tukey's post hoc test. Data are means ± SEM (n = 5). PVH – paraventricular hypothalamic nucleus; CeC – central amygdala; DeN – dorsal endopiriform nucleus; BLA – basolateral amygdaloid nucleus, anterior.
